# Supplementary material for: Study protocol and design for the assessment of paediatric pneumonia from X-ray images using deep learning
Source: BMJ Open. 2021 Apr 8;11(4):e044461. doi: 10.1136/bmjopen-2020-044461 (PMC8039250; doi:10.1136/bmjopen-2020-044461)
Supplement: Author's manuscript [file bmjopen-2020-044461.draft_revisions.pdf]

BMJ Open is committed to open peer review. As part of this commitment we make the peer review history of every article we publish publicly available.

When an article is published we post the peer reviewers' comments and the authors' responses online. We also post the versions of the paper that were used during peer review. These are the versions that the peer review comments apply to.

The versions of the paper that follow are the versions that were submitted during the peer review process. They are not the versions of record or the final published versions. They should not be cited or distributed as the published version of this manuscript.

BMJ Open is an open access journal and the full, final, typeset and author-corrected version of record of the manuscript is available on our site with no access controls, subscription charges or pay-per-view fees (http://bmjopen.bmj.com).

If you have any questions on BMJ Open's open peer review process please email info.bmjopen@bmj.com

BMJ Open

A study protocol and design for the assessment of paediatric pneumonia from X-Ray images using deep learning

Journal:	*BMJ Open*
Manuscript ID	bmjopen-2020-044461
Article Type:	Protocol
Date Submitted by the Author:	13-Sep-2020
Complete List of Authors:	Sun, Mark; Child Health Research Foundation<br>Saha, Senjuti; Child Health Research Foundation, ; Johns Hopkins University Bloomberg School of Public Health,<br>Shah, Syed Ahmar; The University of Edinburgh Usher Institute of Population Health Sciences and Informatics,<br>Luz, Saturnino; University of Edinburgh School of Molecular Genetic and Population Health Sciences, Usher Institute of Population Health Sciences and Informatics<br>Nair, Harish; University of Edinburgh School of Molecular Genetic and Population Health Sciences,<br>Saha, Samir; Child Health Research Foundation; Dhaka Shishu Hospital, Micrbiology
Keywords:	Paediatric radiology < PAEDIATRICS, Respiratory infections < THORACIC MEDICINE, Public health < INFECTIOUS DISEASES, Chest imaging < RADIOLOGY & IMAGING, Diagnostic radiology < RADIOLOGY & IMAGING

SCHOLARONE™
Manuscripts

I, the Submitting Author has the right to grant and does grant on behalf of all authors of the Work (as defined in the below author licence), an exclusive licence and/or a non-exclusive licence for contributions from authors who are: i) UK Crown employees; ii) where BMJ has agreed a CC-BY licence shall apply, and/or iii) in accordance with the terms applicable for US Federal Government officers or employees acting as part of their official duties; on a worldwide, perpetual, irrevocable, royalty-free basis to BMJ Publishing Group Ltd ("BMJ") its licensees and where the relevant Journal is co-owned by BMJ to the co-owners of the Journal, to publish the Work in this journal and any other BMJ products and to exploit all rights, as set out in our licence.

The Submitting Author accepts and understands that any supply made under these terms is made by BMJ to the Submitting Author unless you are acting as an employee on behalf of your employer or a postgraduate student of an affiliated institution which is paying any applicable article publishing charge ("APC") for Open Access articles. Where the Submitting Author wishes to make the Work available on an Open Access basis (and intends to pay the relevant APC), the terms of reuse of such Open Access shall be governed by a Creative Commons licence – details of these licences and which Creative Commons licence will apply to this Work are set out in our licence referred to above.

Other than as permitted in any relevant BMJ Author's Self Archiving Policies, I confirm this Work has not been accepted for publication elsewhere, is not being considered for publication elsewhere and does not duplicate material already published. I confirm all authors consent to publication of this Work and authorise the granting of this licence.

For peer review only

A study protocol and design for the assessment of paediatric pneumonia from X-Ray images using deep learning

Mark George Ford Sun*[1], Senjuti Saha*[1,2,#], Syed Ahmar Shah[5], Saturnino Luz[3,5], Harish Nair[4,5], Samir K Saha[1,4,#]

[1]Child Health Research Foundation, Dhaka, Bangladesh

[2]Johns Hopkins Bloomberg School of Public Health, USA

[3]Centre for Global Health, Usher Institute, University of Edinburgh

[4]Dhaka Shishu (Children) Hospital, Bangladesh

[5]NIHR Global Health Research Unit on Respiratory Health (RESPIRE), Usher Institute, University of Edinburgh, Edinburgh, UK

*These authors made equal contribution to the paper

#Co-correspondence to:

Prof Samir K Saha, PhD, FRCPath
Dhaka Shishu Hospital & Bangladesh Institute of Child Health
Child Health Research Foundation
23/2 Sel Huq Skypark, Block-B Khilji Road,
Dhaka 1207, Bangladesh

Senjuti Saha, PhD
Child Health Research Foundation
23/2 Sel Huq Skypark, Block-B Khilji Road,
Dhaka 1207, Bangladesh
senjutisaha@chrfbd.org; +88 01711646038

samir@chrfbd.org; +8801713461254

Word count: 300 (abstract); 3,248 (main text)

For peer review only

ABSTRACT

Introduction

In low and middle-income countries, pneumonia remains the leading cause of illness and death in children <5 years. The recommended tool for diagnosing paediatric pneumonia is the interpretation of chest X-ray images, which is difficult to standardize and requires trained clinicians/radiologists. Current automated computational tools have primarily focused on assessing adult pneumonia and were trained on images evaluated by a single specialist. We aim to provide a computational tool using a deep-learning approach to diagnose paediatric pneumonia using X-ray images assessed by multiple specialists trained by the WHO expert X-ray image reading panel.

Methods and analysis

Approximately 10,000 paediatric chest X-ray images are currently being collected from an ongoing WHO-supported surveillance study in Bangladesh. Each image will be read by two trained clinicians/radiologists for the presence or absence of primary end-point pneumonia (PEP) in each lung, as defined by the WHO. Images whose PEP labels are discordant in either lung will be reviewed by a third specialist and the final assignment will be made using a majority vote. Convolutional neural networks will be used for lung segmentation to align and scale the images to a reference, and for interpretation of the images for the presence of PEP. The model will be evaluated against an independently collected and labelled set of images from the WHO. The study outcome will be a computational pipeline for the interpretation of chest radiographs for diagnosing pediatric pneumonia.

Ethics and Dissemination

All study protocols were approved by the Ethical Review Committees of the Bangladesh Institute of Child Health, Bangladesh. The study sponsor deemed it unnecessary to attain ethical approval from the Academic and Clinical Central Office for Research and Development of University of Edinburgh, UK. The study uses existing X-ray images from an ongoing WHO-funded surveillance. All findings will be published in an open-access journal.

For peer review only

Strengths and limitations of this study

- This manuscript describes the study protocol and design for the assessment of paediatric pneumonia from X-Ray images using deep learning.
- The main strength of the study is that it will employ a large, high confidence dataset of ~10,000 paediatric chest X-ray images interpreted by multiple trained readers for the presence of primary endpoint pneumonia.
- Another strength is that the computational tool will be evaluated against two external paediatric chest X-ray image datasets to assess generalizability of the model.
- The primary limitation of the study is that the computational model will be trained from chest X-ray images derived from a single study site.

For peer review only

INTRODUCTION

Paediatric pneumonia overview

Pneumonia is a leading cause of childhood illness and death in developing countries. Globally, about 138 million cases and 0.9 million deaths are estimated to occur annually due to pneumonia [1] with about six million cases in Bangladesh alone [1]. However, determining the disease prevalence is complicated due to the lack of reliable diagnostic tools: 1) blood cultures are insensitive as most cases are likely not bacteremic [2] and 2) other diagnostic procedures are difficult to standardize. While the true proportions of pneumonia etiologies (viral/bacterial) are relatively unknown, 34.2% of Bangladesh pneumonia patients receive empirical antibiotics [3]. While image based diagnostics such as the interpretation of frontal chest X-ray images to identify paediatric pneumonia are regarded as the "gold standard" [4], they are difficult to standardize and the interpretations are often subjective, differing between radiologists/clinicians [5,6]. Furthermore, the limited number of well-trained radiologists/clinicians restricts the use of X-ray images as a ubiquitous diagnostic tool for pneumonia. This lack of accurate diagnosis hampers the institution of evidence-based intervention strategies and vaccine impact measurements.

Deep learning approaches have successfully been applied to assist in the detection and classification of human diseases like breast cancer [7], tuberculosis [8], diabetic retinopathy [9] and pneumonia [10–12] directly from images. In some instances, these systems performed as well as trained health professionals. Our goal is to construct a computational framework to automatically and systematically interpret paediatric chest X-ray images to diagnose pneumonia. Such an automated diagnostic system, if successful, will accelerate the adoption of chest X-ray images for evidence-based case management and contribute to the standardization of X-ray image analysis. This will encourage clinicians to rationalise the use of antibiotic therapies and to facilitate antibiotic stewardship.

Diagnosing paediatric pneumonia

Chest X-ray images interpreted to have primary endpoint pneumonia (PEP) are considered the "gold standard" for the diagnosis of paediatric pneumonia. The PEP case definition was

For peer review only

developed by the WHO as part of clinical trials to assess the efficacy of vaccines against *Streptococcus pneumoniae* and *Haemophilus influenza* type b (Hib) and is defined as the presence of end-point consolidation or pleural effusion [4]. Despite efforts to standardize the interpretation of chest radiographs by training specialists to the WHO definitions, significant discrepancies are still observed between readers interpreting the same chest X-ray images, due to the subjective nature of the diagnosis [5,6]. These discrepancies may result in studies using a radiological definition of pneumonia to underestimate its true burden [13]. To overcome differences between trained readers, a panel of multiple readers may be employed to interpret chest X-ray images for the presence of pneumonia [6].

Establishing well-funded and rigorous clinical trial, requiring multiple trained readers to evaluate every chest X-ray image is an onerous task. To this end, groups have leveraged natural language processing (NLP) systems to analyze radiology reports from clinical settings to programmatically assign labels, enabling them to create large collections of labelled chest X-ray images for the presence of pneumonia in adult lungs. These large training datasets (40,000 – 110,000 images) were used to train deep learning computational models to diagnose pneumonia directly from chest X-ray images [10,11]. Zech and colleagues found that models trained on adult chest X-ray images evaluated by a single reader achieved a performance of 0.815 area under the receiver operator curve (AUROC) on an external dataset independently collected from the training dataset. Performance on images collected from the same data source was 0.931 AUROC [11]. In light of the grading discrepancies found between readers interpreting pediatric chest X-ray images for pneumonia, the proposed study will use multiple readers trained by the expert WHO X-ray image reading panel. Additionally, all readers will be blinded to the labels provided by the other readers. When multiple readers are leveraged to diagnose pneumonia, the study size is smaller than those used by NLP systems. Mahomed and colleagues used 858 pediatric chest X-ray images interpreted for PEP by three readers to train a support vector machine (SVM) for classification. Performance on images collected from the same data source was 0.850 AUROC [12].

For peer review only

Objectives

The primary project aims are (1) to collect and label ~10,000 paediatric chest X-rays images with high confidence, indicating if the right and left lung are healthy or if either lung meets the primary-end point pneumonia criteria and (2) to develop a software tool leveraging advances in the field of image recognition, in particular deep learning architectures such as DenseNet [14], ResNet [15,16], and Inception [17,18] and in model training regimes, to diagnose paediatric pneumonia from chest X-ray images and (3) to evaluate the model against 130 independently collected images from sites differing from those described in this study and labelled by the WHO Chest Radiography in Epidemiological Studies (WHO CRES) working group [19].

METHODS AND ANALYSIS

Study design

The study proposes to develop an automated software tool to diagnose paediatric pneumonia directly from chest X-ray images. The X-ray images will be collected and labelled by multiple radiologists and clinicians trained in reading and interpreting X-rays by the WHO expert panel. This image dataset will be used to train a computational model and to develop a pipeline to diagnose paediatric pneumonia. The software tool will be evaluated against an image dataset independently collected from a different study site and labelled by the WHO CRES working group (Figure 1) [20].

Participants and data source

Chest X-ray images used to train the computational model will be derived from the on-going WHO-funded Invasive Bacterial Vaccine Preventable Diseases (IB-VPD) Surveillance Platform in Bangladesh [21,22], which were performed on the advice of the treating physician. This surveillance system monitors pneumonia, meningitis, and sepsis in children under 5 years old in-patients, using protocols described elsewhere [23].

The project is on-going. Collection of the chest X-ray images started in 2013 and we expect to reach 10,000 chest X-ray images that meet the inclusion criteria in 2021.

For peer review only

Images being collected for model training (we expect to collect ~ 10 000 images) will be obtained from Dhaka Shishu Hospital (DSH), where our group has been systematically storing digital X-rays since 2013 from children with suspected pneumonia. Two machines are used to capture the images: 1) SG Healthcare Jumong General and 2) TRIUP International Corp TRF100. The intent is for 100 images derived from the Kumudini Women's Medical College and Hospital (KWMCH), also an IB-VPD Surveillance Platform partner, to be included as a test set to assess the impact of images collected from different X-ray machines. The KWMCH site has two machines to capture images: 1) Hitachi Radnext 50 and 2) General Electric Dx-525. In the IB-VPD surveillance [23], written consent is obtained from parents or caregivers of all participants for other aspects of the study, including collection of data and additional analysis. Images of any participants that withdraw the consent from the surveillance study will not be considered for this study. The data generated from the individual patients will be deidentified prior to analysis.

Inclusion Criteria

An X-ray image will be included in this study if

- The age of the child for whom the X-ray is performed is <60 months,
- The X-ray was performed in one of the two study hospitals and the child was enrolled in the IB-VPD surveillance study, and
- The X-ray image captures the lung area

The inclusion criteria for the IB-VPD surveillance system for pneumonia are:

- The age of the child is <60 months,
- The child had coughing or difficulty breathing and tachypnea when calm at a rate of ≥60 breaths/min in an infant aged <2 months, or
- ≥50 breaths/min in an infant aged 2 to <12 months, or ≥40 breaths/min in children ≥12 months

Exclusion Criteria

Any image that is marked "uninterpretable" (features of the images are not interpretable with respect to presence or absence of primary end-point pneumonia) by the first two readers.

For peer review only

Sample size

In this study, we are attempting to train computational models rather than to assess if two populations differ. For this reason, we turn to literature to estimate the training dataset sizes required to build the proposed software tool to interpret paediatric chest X-rays. The tool to diagnose paediatric pneumonia (objective 2) will be composed of two stages: 1) lung detection to center, scale, and rotation align the chest X-ray images and 2) interpretation of paediatric chest X-rays for the presence of pneumonia.

To identify the location of lungs within the X-ray image, Dai and colleagues developed a generative adversarial network (GAN) and a baseline residual block network. They report Dice coefficient values of 97.3% and 96.3% respectively, where the Dice coefficient is a measure of similarity between the predicted location and a reference mask, with just 247 adult chest X-rays images [24]. The results will likely translate to the proposed study as we are interested in the identification of the same gross anatomical structures. This suggests that detecting the lung's location within paediatric chest X-rays can readily be achieved with training using only a small proportion (about 300 from 10,000) of the collected chest X-ray images.

For interpretation of chest X rays, we'll focus on studies that used expert panels to label X-ray images as a means to estimate the dataset size required to train a neural network to diagnose paediatric pneumonia directly from chest X-ray images. Prior studies used natural language processing (NLP) systems to analyze radiology reports to programmatically assign labels, enabling large collections of chest X-ray images to be labelled [10,11]. However, due to the variability found between trained readers when diagnosing paediatric pneumonia from chest X-ray images [5,6], we are of the opinion that for this study a more appropriate strategy is to have each image assessed by multiple readers to ensure a high quality training dataset. Recent deep learning methods have diagnosed diseases such as tuberculosis after training on 1,007 chest X-ray images with a performance of 0.99 for the area under the receiver operator curve (AUROC) [8] and diabetic retinopathy with a performance of 0.974 AUROC using 128,175 retinal training images (28.1% were classified as referable diabetic retinopathy) [9]. For the diagnosis of referable diabetic retinopathy, [6]Gulshan and colleagues found that using 10,000 retinal images to train their model resulted in a sensitivity and specificity respectively of 97% and 75%

For peer review only

with saturation occurring at 60,000 images with a sensitivity and specificity respectively of 97.5% and 93.4%. We noted that the performance found between a reading panel and images labelled by an expert reader with a sensitivity and specificity respectively of 77% and 96.3% for paediatric pneumonia [6].

To date 8,002 paediatric chest X-ray images have been reviewed by a single clinician, of which 2,320 (29%) were identified with primary end-point pneumonia in either lung, 4786 (60%) had no pneumonia, and 898 (11%) were uninterpretable. We expect this class ratio to remain true for the complete dataset. Together, these studies and observations suggest that the 10,000 paediatric chest X-rays being collected will result in a suitable dataset to train a neural network to identify pneumonia cases among paediatric chest X-ray images.

Study Procedure

To build a software tool capable of diagnosing paediatric pneumonia directly from X-ray images, it is first necessary to establish a representative training dataset (Objective 1) that is of a sufficiently large size for a computational model to be successfully trained. To this end, ~10,000 paediatric chest X-rays will be collected and labelled by radiologists / clinicians standardized to the WHO definitions (each blinded to all labels provided by the other readers) [4] who will interpret the images as having 1) primary end-point pneumonia (PEP), 2) other lung infiltrates, and/or 3) pleural fluid in either the left or right lung, for a total of six possible binary outcomes. By having multiple labels for both lungs rather than a single binary value indicating if the chest X-ray image has a pneumonia lung will enable information from both lungs to be leveraged and information about other non-PEP classes may be used to guide the pneumonia prediction task. A challenge to assigning a pneumonia label to an image is the high discordance observed between diagnoses from trained radiologists / clinicians [5]. We plan on mitigating this by having multiple readers standardized to the WHO definitions to read each chest X-ray image. Specifically, two primary readers will assess all images meeting the described inclusion criteria. Chest X-ray images having discordant PEP labels between the two readers in either the right or left lung, when considering the lungs independently, will be reassessed by a third reader. A simple majority voting will be used to assign the multiclass labels for images. The remaining discordant labels (ie. not PEP) will be assigned randomly to reflect uncertainty in the label.

For peer review only

234 Since “uninterpretable” images may still have evidence of some of the six outcomes, images labelled as “uninterpretable” by both readers will be excluded from the computational modeling process. Images labelled as “uninterpretable” by one primary reader but “interpretable” by the other will be re-evaluated by the third reader.

238 The software tool to diagnose paediatric pneumonia (objective 2) will be composed of two stages: 1) lung detection to center, scale, and rotation align the chest X-ray images and 2) interpretation of paediatric chest X-ray images for the presence of pneumonia. Chest X-ray images of paediatric patients can be highly variable, specifically in resource poor settings without well-trained personnel and/or high patient flow, ranging from images of uncentered whole child bodies to images of the only whole lung (Figure 2). To aid the paediatric pneumonia prediction task, we first aim to identify the location of the lung. While techniques such as data augmentation can minimize the impact of translation, scaling, and rotation on a neural network’s performance, explicitly removing these sources of variation can improve performance [25]. We will evaluate two approaches, the first is a variant of the VGG (Visual Geometry Group) architecture with residual network blocks for pixel-level object detection. This has previously been shown to work well for adult lung segmentation [24,26], in combination with image histogram equilibration. The lung detection model will initially be trained on 300 paediatric chest X-ray images and evaluated on a validation set of an additional 100 paediatric chest X-ray images and assessed using the Dice coefficient to evaluate the model’s performance. Additional images may be used if the model’s performance is deemed insufficient. In line with observations by Dai and colleagues [24], 300 images should be sufficient for our lung segmentation and centering task. Key point detection on the segmented images by BRISK [27] or FREAK [28] to align the images to a reference image using OpenCV [29]. The alternative approach will be to explore the use of spatial transformer networks [25] in conjunction with object recognition deep learning architectures, thereby enabling a single model to detect the lung and diagnose paediatric pneumonia.

260 The second stage of the software tool will diagnose pneumonia from the centered and scaled paediatric chest X-ray images. This identification task will be performed in a multi-task prediction setting, namely that the computational model’s goal will be to predict 1) primary

end-point pneumonia, 2) other lung infiltrates, and/or 3) pleural fluid in both the left and right lung for a total of 6 binary labels. The binary cross entropy loss function will be used during model training. An ensemble of five models will form the final predictor, whose predictions for each of the 6 output classes will be the unweighted average of the five models' predicted class probabilities.

Performance Evaluation

To select the model hyper-parameters, the data will be partitioned into 5-folds. This will allow us to train models on 80% and validate on 20% of the data and to compare models with different hyper-parameters. Hyper-parameters will be determined with a mixture of grid searching and prior literature values. Performance stability for a given set of model hyper-parameters will be made using 5-fold cross-validation. These five models will form an ensemble that will form the final predictions for each of the 6 output classes by taking the unweighted average of the predicted class probabilities for each label.

Two image datasets (objective 3) will be used to evaluate the software tool. The first test set will be from KWMCH, whose labels will be provided by the same readers who labelled the training image dataset. This dataset will enable the study to evaluate the impact of using different X-ray imagining machines and technicians and disease prevalence. The second dataset will be from WHO CRES, whose images were independently collected and independently labelled by clinicians and radiologists following the WHO Radiological Pneumonia definitions. This will assess how transferable our method will be to other study sites.

Ethics and dissemination

All X-rays are performed as part of routine clinical care at the attending physician's discretion. Written consent is obtained from parents or caregivers of all participants for other aspects of the study, including collection of data and additional analysis. Ethical approval was obtained from the Bangladesh Institute of Child Health, Dhaka Shishu Hospital, Bangladesh. The study sponsor deemed it unnecessary to attain ethical approval from the Academic and Clinical Central Office for Research and Development (ACCORD) of University of Edinburgh, UK.

For peer review only

All x-ray labels and statistical codes will be made openly available for all on github. The model and images will be made available upon request. All publications will be open access. Patients and the public were involved in the design and dissemination plans for our research.

Patient and Public Involvement

No patient involved.

DISCUSSION

The study will be the first to use a large dataset whose labels are derived from multiple expert readers to train a deep-learning model to diagnose paediatric primary-end point pneumonia. Prior work to identify pneumonia in adult lungs using a computational model achieved a performance of 0.815 AUROC on an independently collected test set. This computational model was trained on chest X-ray images labelled from a single reader whose pneumonia labels were extracted from radiology reports via a NLP system that may introduce extraction errors [11]. We are expecting our model to have similar or better performance since the training data is evaluated by multiple readers and the goal of diagnosing primary-end point pneumonia is better defined.

A limitation of the study is the use of chest X-ray images from individuals younger than 60 months of age derived from a single institution to construct a computational model to interpret chest X-ray images for the presence of pneumonia. Findings from the study may not generalize to individuals older than or equal to 60 months of age. However, this is the most appropriate age group for this study as more than 90% of pediatric pneumonia cases are limited to children <5 years of age.

While the computational model will be evaluated against an independently collected and labelled chest X-ray image dataset for the presence or absence of primary end-point pneumonia (WHO CRES dataset), some findings may not generalize to other settings (as the training data is from a single institution). To overcome this limitation, we are aiming to extend this study as a multi-country surveillance, if successful.

For peer review only

CONCLUSION

The primary tool for diagnosing paediatric pneumonia is the interpretation of chest X-ray images. Two barriers restrict its use as a ubiquitous diagnostic tool. The first is the observed discrepancies found between readers when interpreting images requiring a panel of readers to ensure the correct diagnosis is made for epidemiological studies. The second is the limited number of trained radiologists/clinicians. A computational tool that can systematically diagnose paediatric pneumonia directly from chest X-ray images will enable studies to broadly use chest X-ray images, which include clinical trials, monitoring the impact of vaccines interventions, and facilitation of antibiotic stewardship by providing evidence-based patient management.

Acknowledgements

This research was commissioned by the UK National Institute for Health Research (NIHR) Global Health Research Unit on Respiratory Health (RESPIRE), using UK Aid from the UK Government. The views expressed in this publication are those of the author(s) and not necessarily those of the NIHR or the UK Department of Health and Social Care. We are grateful to Mohammad Shahidul Islam and Md. Shariful Islam for the intellectual and technical guidance

Author's Contributors

Conceptual construction of research and design: MGFS, SS, and SKS. Critical manuscript review: MGFS, SS, SKS, SAS, SLF. All authors have approved the final version of the manuscript for submission for publication.

Funding

This work has received funding support from NIHR Global Health Research Unit on Respiratory Health (RESPIRE) 16/136/109.

Competing interests

None declared

For peer review only

Ethics

All study protocols were approved by the Ethical Review Committees of the Bangladesh Institute of Child Health.

References

1 McAllister DA, Liu L, Shi T, *et al.* Global, regional, and national estimates of pneumonia morbidity and mortality in children younger than 5 years between 2000 and 2015: a systematic analysis. *Lancet Glob Health* 2019;**7**:e47–e57.

2 O'Brien KL, Baggett HC, Brooks WA, *et al.* Causes of severe pneumonia requiring hospital admission in children without HIV infection from Africa and Asia: the PERCH multi-country case-control study. *The Lancet* 2019.

3 National Institute of Population Research and Training. BANGLADESH DEMOGRAPHIC AND HEALTH SURVEY. 2014.https://dhsprogram.com/pubs/pdf/FR311/FR311.pdf

4 Cherian T, Mulholland EK, Carlin JB, *et al.* Standardized interpretation of paediatric chest radiographs for the diagnosis of pneumonia in epidemiological studies. *Bull World Health Organ* 2005;**83**:353–359.

5 Elemraid MA, Muller M, Spencer DA, *et al.* Accuracy of the interpretation of chest radiographs for the diagnosis of paediatric pneumonia. *PLoS One* 2014;**9**:e106051.

6 McCollum ED, Ahmed S, Chowdhury NH, *et al.* Chest radiograph reading panel performance in a Bangladesh pneumococcal vaccine effectiveness study. *BMJ Open Respir Res* 2019;**6**:e000393.

7 Liu Y, Gadepalli K, Norouzi M, *et al.* Detecting cancer metastases on gigapixel pathology images. *ArXiv Prepr ArXiv170302442* 2017.

8 Lakhani P, Sundaram B. Deep learning at chest radiography: automated classification of pulmonary tuberculosis by using convolutional neural networks. *Radiology* 2017;**284**:574–582.

9 Gulshan V, Peng L, Coram M, *et al.* Development and validation of a deep learning algorithm for detection of diabetic retinopathy in retinal fundus photographs. *Jama* 2016;**316**:2402–2410.

10 Rajpurkar P, Irvin J, Zhu K, *et al.* Chexnet: Radiologist-level pneumonia detection on chest x-rays with deep learning. *ArXiv Prepr ArXiv171105225* 2017.

For peer review only

11 Zech JR, Badgeley MA, Liu M, *et al.* Variable generalization performance of a deep learning model to detect pneumonia in chest radiographs: A cross-sectional study. *PLoS Med* 2018;**15**:e1002683.

12 Mahomed N, van Ginneken B, Philipsen RHHM, *et al.* Computer-aided diagnosis for World Health Organization-defined chest radiograph primary-endpoint pneumonia in children. *Pediatr Radiol* 2020;**50**:482–91. doi:10.1007/s00247-019-04593-0

13 Madhi SA, Klugman KP. World Health Organisation definition of "radiologically-confirmed pneumonia" may under-estimate the true public health value of conjugate pneumococcal vaccines. *Vaccine* 2007;**25**:2413–9. doi:10.1016/j.vaccine.2006.09.010

14 Huang G, Liu Z, Van Der Maaten L, *et al.* Densely connected convolutional networks. In: *Proceedings of the IEEE conference on computer vision and pattern recognition*. 2017. 4700–4708.

15 He K, Zhang X, Ren S, *et al.* Identity mappings in deep residual networks. In: *European conference on computer vision*. Springer 2016. 630–645.

16 He K, Zhang X, Ren S, *et al.* Deep residual learning for image recognition. In: *Proceedings of the IEEE conference on computer vision and pattern recognition*. 2016. 770–778.

17 Szegedy C, Liu W, Jia Y, *et al.* Going deeper with convolutions. In: *Proceedings of the IEEE conference on computer vision and pattern recognition*. 2015. 1–9.

18 Szegedy C, Vanhoucke V, Ioffe S, *et al.* Rethinking the inception architecture for computer vision. In: *Proceedings of the IEEE conference on computer vision and pattern recognition*. 2016. 2818–2826.

19 WHO Chest Radiography in Epidemiological Studies (CRES). https://who-cres.mcri.edu.au/

20 Mahomed N, Fancourt N, De Campo J, *et al.* Preliminary report from the World Health Organisation chest radiography in epidemiological studies project. *Pediatr Radiol* 2017;**47**:1399–1404.

21 Hasan AZ, Saha S, Saha SK, *et al.* Using pneumococcal and rotavirus surveillance in vaccine decision-making: A series of case studies in Bangladesh, Armenia and the Gambia. *Vaccine* 2018;**36**:4939–43. doi:10.1016/j.vaccine.2018.06.001

22 Global Invasive Bacterial Vaccine-Preventable Diseases Surveillance — 2008–2014. https://www.cdc.gov/mmwr/preview/mmwrhtml/mm6349a4.htm (accessed 2 Sep 2020).

23 Saha SK, Hossain B, Islam M, *et al.* Epidemiology of invasive pneumococcal disease in Bangladeshi children before introduction of pneumococcal conjugate vaccine. *Pediatr Infect Dis J* 2016;**35**:655–661.

For peer review only

24 Dai W, Dong N, Wang Z, *et al.* Scan: Structure correcting adversarial network for organ segmentation in chest x-rays. In: *Deep Learning in Medical Image Analysis and Multimodal Learning for Clinical Decision Support*. Springer 2018. 263–273.

25 Jaderberg M, Simonyan K, Zisserman A, *et al.* Spatial transformer networks. In: *Advances in neural information processing systems*. 2015. 2017–2025.

26 Bansal A, Chen X, Russell B, *et al.* Pixelnet: Towards a general pixel-level architecture. *ArXiv Prepr ArXiv160906694* 2016.

27 Leutenegger S, Chli M, Siegwart RY. BRISK: Binary robust invariant scalable keypoints. In: *2011 International conference on computer vision*. Ieee 2011. 2548–2555.

28 Alahi A, Ortiz R, Vandergheynst P. Freak: Fast retina keypoint. In: *2012 IEEE Conference on Computer Vision and Pattern Recognition*. Ieee 2012. 510–517.

29 Bradski G. The OpenCV Library. *Dr Dobbs J Softw Tools* 2000.

FIGURE LEGENDS

Figure 1: Study design schema. PEP: Primary end point pneumonia; KWMCH : Kumudini Women's Medical College Hospital

Figure 2: Digitized chest X-ray images include (Left) full body images and (Right) images of just the lung area.

For peer review only

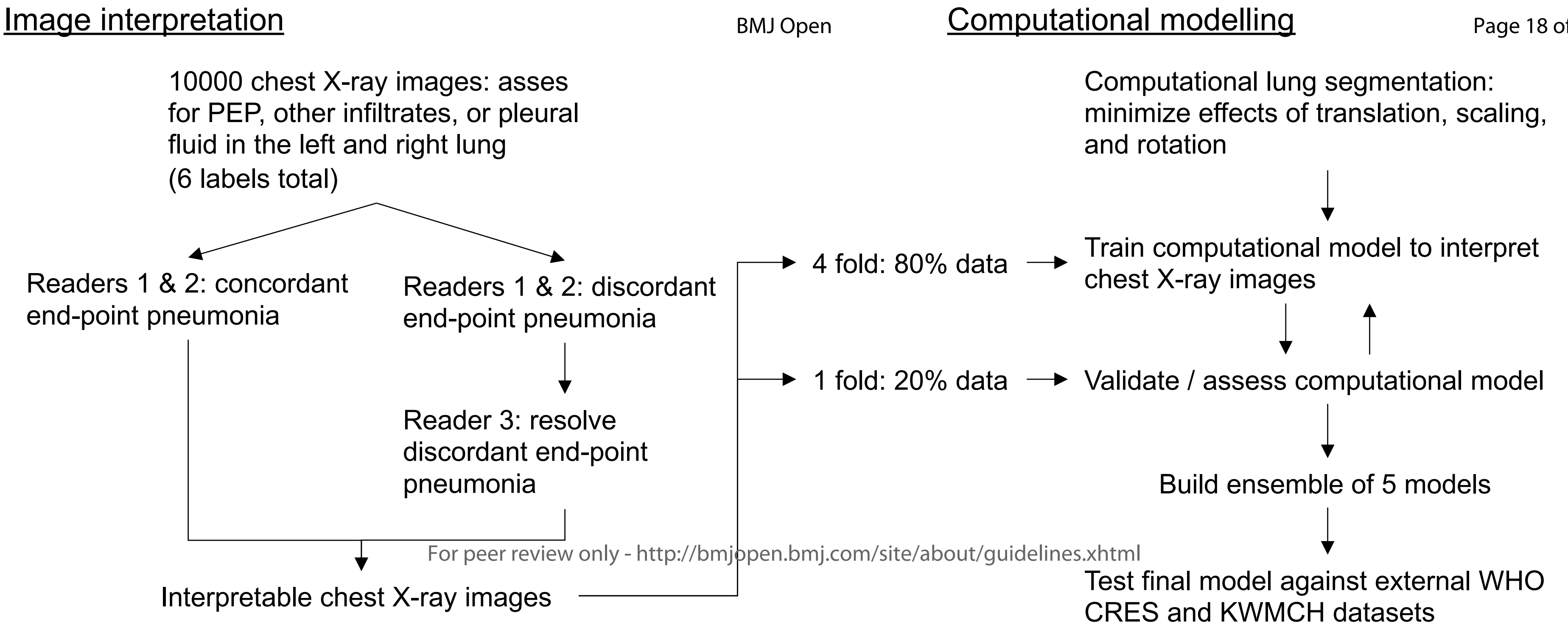

For peer review only - http://bmjopen.bmj.com/site/about/guidelines.xhtml

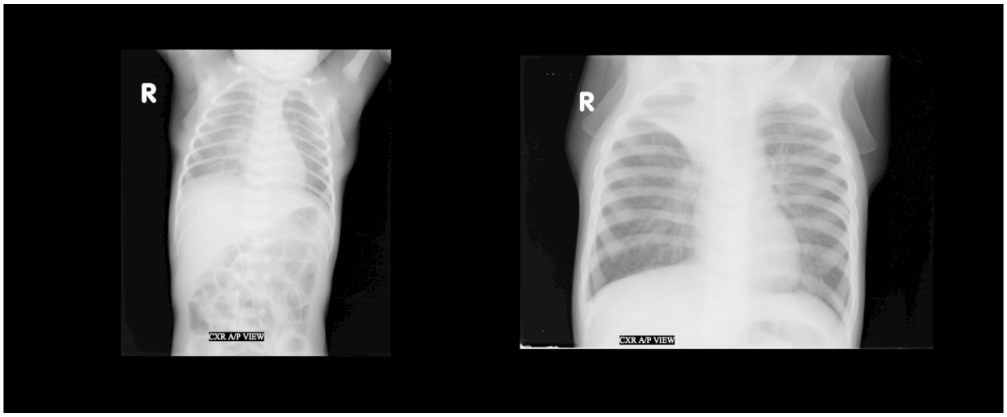

Figure 2: Digitized chest X-ray images include (Left) full body images and (Right) images of just the lung area.

BMJ Open

A study protocol and design for the assessment of paediatric pneumonia from X-Ray images using deep learning

Journal:	*BMJ Open*
Manuscript ID	bmjopen-2020-044461.R1
Article Type:	Protocol
Date Submitted by the Author:	30-Dec-2020
Complete List of Authors:	Sun, Mark; Child Health Research Foundation<br>Saha, Senjuti; Child Health Research Foundation; Johns Hopkins University Bloomberg School of Public Health,<br>Shah, Syed Ahmar; The University of Edinburgh Usher Institute of Population Health Sciences and Informatics,<br>Luz, Saturnino; University of Edinburgh School of Molecular Genetic and Population Health Sciences, Usher Institute of Population Health Sciences and Informatics<br>Nair, Harish; University of Edinburgh School of Molecular Genetic and Population Health Sciences,<br>Saha, Samir; Child Health Research Foundation
**Primary Subject Heading**:	Diagnostics
Secondary Subject Heading:	Diagnostics, Health informatics, Infectious diseases, Public health, Respiratory medicine
Keywords:	Paediatric radiology < PAEDIATRICS, Respiratory infections < THORACIC MEDICINE, Public health < INFECTIOUS DISEASES, Chest imaging < RADIOLOGY & IMAGING, Diagnostic radiology < RADIOLOGY & IMAGING

SCHOLARONE™
Manuscripts

I, the Submitting Author has the right to grant and does grant on behalf of all authors of the Work (as defined in the below author licence), an exclusive licence and/or a non-exclusive licence for contributions from authors who are: i) UK Crown employees; ii) where BMJ has agreed a CC-BY licence shall apply, and/or iii) in accordance with the terms applicable for US Federal Government officers or employees acting as part of their official duties; on a worldwide, perpetual, irrevocable, royalty-free basis to BMJ Publishing Group Ltd ("BMJ") its licensees and where the relevant Journal is co-owned by BMJ to the co-owners of the Journal, to publish the Work in this journal and any other BMJ products and to exploit all rights, as set out in our licence.

The Submitting Author accepts and understands that any supply made under these terms is made by BMJ to the Submitting Author unless you are acting as an employee on behalf of your employer or a postgraduate student of an affiliated institution which is paying any applicable article publishing charge ("APC") for Open Access articles. Where the Submitting Author wishes to make the Work available on an Open Access basis (and intends to pay the relevant APC), the terms of reuse of such Open Access shall be governed by a Creative Commons licence – details of these licences and which Creative Commons licence will apply to this Work are set out in our licence referred to above.

Other than as permitted in any relevant BMJ Author's Self Archiving Policies, I confirm this Work has not been accepted for publication elsewhere, is not being considered for publication elsewhere and does not duplicate material already published. I confirm all authors consent to publication of this Work and authorise the granting of this licence.

For peer review only

A study protocol and design for the assessment of paediatric pneumonia from X-Ray images using deep learning

Mark George Ford Sun*[1], Senjuti Saha*[1,2,#], Syed Ahmar Shah[5], Saturnino Luz[3,5], Harish Nair[4,5], Samir K Saha[1,4,#]

[1]Child Health Research Foundation, Dhaka, Bangladesh

[2]Johns Hopkins Bloomberg School of Public Health, USA

[3]Centre for Global Health, Usher Institute, University of Edinburgh

[4]Dhaka Shishu (Children) Hospital, Bangladesh

[5]NIHR Global Health Research Unit on Respiratory Health (RESPIRE), Usher Institute, University of Edinburgh, Edinburgh, UK

*These authors made equal contribution to the paper

#Co-correspondence to:

Prof Samir K Saha, PhD, FRCPath
Dhaka Shishu Hospital & Bangladesh Institute of Child Health
Child Health Research Foundation
23/2 Sel Huq Skypark, Block-B Khilji Road,
Dhaka 1207, Bangladesh
samir@chrfbd.org; +8801713461254

Senjuti Saha, PhD
Child Health Research Foundation
23/2 Sel Huq Skypark, Block-B Khilji Road,
Dhaka 1207, Bangladesh
senjutisaha@chrfbd.org; +88 01711646038

Word count: 299 (abstract); 3,944 (main text)

For peer review only

ABSTRACT

Introduction

In low and middle-income countries, pneumonia remains the leading cause of illness and death in children <5 years. The recommended tool for diagnosing paediatric pneumonia is the interpretation of chest X-ray images, which is difficult to standardize and requires trained clinicians/radiologists. Current automated computational tools have primarily focused on assessing adult pneumonia and were trained on images evaluated by a single specialist. We aim to provide a computational tool using a deep-learning approach to diagnose paediatric pneumonia using X-ray images assessed by multiple specialists trained by the WHO expert X-ray image reading panel.

Methods and analysis

Approximately 10,000 paediatric chest X-ray images are currently being collected from an ongoing WHO-supported surveillance study in Bangladesh. Each image will be read by two trained clinicians/radiologists for the presence or absence of primary end-point pneumonia (PEP) in each lung, as defined by the WHO. Images whose PEP labels are discordant will be reviewed by a third specialist and the final assignment will be made using a majority vote. Convolutional neural networks will be used for lung segmentation to align and scale the images to a reference, and for interpretation of the images for the presence of PEP. The model will be evaluated against an independently collected and labelled set of images from the WHO. The study outcome will be an automated method for the interpretation of chest radiographs for diagnosing pediatric pneumonia.

Ethics and Dissemination

All study protocols were approved by the Ethical Review Committees of the Bangladesh Institute of Child Health, Bangladesh. The study sponsor deemed it unnecessary to attain ethical approval from the Academic and Clinical Central Office for Research and Development of University of Edinburgh, UK. The study uses images from an ongoing WHO-coordinated surveillance. All X-ray labels and codes will be made openly available. The model and images will be made available upon request.

For peer review only

Strengths and limitations of this study

- The main strength of the study is that it will employ a large, high confidence dataset of ~10,000 paediatric chest X-ray images interpreted by multiple trained readers for the presence of primary endpoint pneumonia.
- Another strength is that the computational tool will be evaluated against two external paediatric chest X-ray image datasets to assess generalizability of the model.
- The primary limitation of the study is that the computational model will be trained from chest X-ray images derived from a single study site.

For peer review only

INTRODUCTION

Paediatric pneumonia overview

Pneumonia is a leading cause of childhood illness and death in developing countries. Globally, about 138 million cases and 0.9 million deaths are estimated to occur annually due to pneumonia with about six million cases in Bangladesh alone [1]. However, determining the disease prevalence is complicated due to the lack of reliable diagnostic tools: 1) blood cultures are insensitive as most cases are likely not bacteremic and therefore a blood culture cannot determine a bacterial respiratory infection [2] and 2) other diagnostic procedures like radiological findings are difficult to standardize. While the true proportions of pneumonia etiologies (viral/bacterial) are unknown [2], 34% of children in Bangladesh diagnosed with pneumonia receive empirical antibiotics [3]. Image based diagnostics such as the interpretation of chest X-ray images to identify paediatric pneumonia are regarded as the "gold standard" [4], they are difficult to standardize and the interpretations can vary between radiologists and clinicians [5,6]. Furthermore, the limited number of well-trained radiologists and clinicians limits the use of X-ray images as a ubiquitous diagnostic tool for pneumonia. This lack of accurate diagnosis hampers the determination of the true burden of pneumonia, and the proportion that is preventable by vaccination, which in turn impedes institution of evidence-based intervention strategies and vaccine impact measurements.

Deep learning methods such as convolutional neural networks (CNNs) may be used to expedite the adoption of chest X-ray images for the diagnosis of pneumonia. CNNs are composed of multiple feature extractors stacked on top of each other. During the training process the feature extractors near the model input learn primitive image features such as edges, whereas feature extractors deeper into the model learn domain specific relationships important for the diagnostic task. CNNs have successfully been applied to assist in the detection and classification of human diseases like breast cancer [7], tuberculosis [8], diabetic retinopathy [9] and pneumonia [10–12] directly from images. In some instances, these systems performed as well as trained health professionals. Our goal is to construct a computational framework to

For peer review only

automatically and systematically interpret paediatric chest X-ray images to diagnose pneumonia. If successful, an automated system to detect pediatric pneumonia will have significant public health benefits. It will accelerate the adoption of chest X-ray images in low- and middle-income country (LMIC) settings where there is a lack of trained radiologists and clinicians and contribute to the standardization of X-ray image analysis. This will encourage clinicians to rationalise the use of antibiotic therapies and to facilitate antibiotic stewardship.

Diagnosing paediatric pneumonia

Chest X-ray images interpreted to have primary endpoint pneumonia (PEP) are considered the "gold standard" for the diagnosis of paediatric pneumonia [4]. The PEP case definition was developed by the WHO as part of clinical trials to assess the efficacy of vaccines against *Streptococcus pneumoniae* and *Haemophilus influenza* type b (Hib) and is defined as the presence of end-point consolidation or pleural effusion [4]. Despite efforts to standardize the interpretation of chest radiographs by training specialists to the WHO definitions, significant discrepancies are still observed between readers interpreting the same chest X-ray images, due to the subjective nature of the diagnosis [5,6]. These discrepancies may result in studies using a radiological definition of pneumonia to underestimate its true burden [13]. To overcome differences between trained readers, a panel of multiple readers may be employed to interpret images [6].

Establishing well-funded and rigorous clinical trials requires multiple trained readers to evaluate every chest X-ray image is an onerous task. This time intensive task may be alleviated with the aid of a computational tool that can diagnose pneumonia directly from chest X-ray images. CNN models are one such tool, but for many problem domains require thousands of labelled images to successfully be trained from a randomly initialized state. To this end, groups have leveraged natural language processing (NLP) systems to analyze radiology reports from clinical settings to programmatically assign labels, enabling them to create large collections of labelled chest X-ray images for the presence or absence of pneumonia in adult lungs. These large training datasets

For peer review only

(40,000 – 110,000 images) were evaluated by a single reader and used to train CNN models to diagnose pneumonia directly from chest X-ray images [10,11]. Transfer learning can be applied to reduce model training time or enable model training should the dataset be too small to train a model from a randomly initialized state. Models are first pretrained on a large image dataset such as ImageNet [14] that contain more than a million labelled images of common entities. These pretrained models are subsequently transferred by finetuning the model to the domain of interest [15]. Zech and colleagues found that using transfer learning for a DenseNet-121 model finetuned on a large dataset of adult chest X-ray images whose diagnosis were derived from radiology reports achieved a performance of 0.931 for the area under the receiver operator curve (auROC). When evaluated on an independently collected external benchmark dataset, the model performed significantly worse with an auROC of 0.815 [11].

Efforts to increase performance have involved the development of larger CNN models through the use of model ensembles, whose models have different architectures. As the choice of an architecture restricts the functions that can be learned to associate an X-ray image to a diagnosis label, using multiple different architectures may expand the space of functions that can be learned. Chouhan and colleagues used transfer learning with five different models [16] based on the AlexNet [17], DenseNet-121 [18], Inception V3 [19], GoogLeNet [20], and ResNet18 [21] architectures, for a pediatric pneumonia Xray image dataset containing 5856 labelled images [22]. Combining the individual model predictions by a majority vote (99.34 auROC) [16] or in a linear combination scheme (99.76 auROC) [23] were suggestive that a performance increase relative to a single model is possible. Zech and colleagues found that a DenseNet-121 model finetuned with images from a single site in 3 of 5 instance had a reduced performance on a benchmark dataset collected from another study site [11]. Since the ensemble was evaluated on images from the same study site, without further investigation it is unclear if the performance gains are a result of the ensemble exploiting confounding features of the study site or learning discriminatory radiological features of pneumonia.

For peer review only

During CNN model training, image features responsible for the radiological diagnosis of pneumonia are learned. Rather than learning the image features directly from the training dataset, the images features may be explicitly specified. Ke and colleagues used global image descriptors of hue, saturation, and brightness as input to a fully connected neural network to predict pneumonia [24]. Representative pixels of disease lung tissue were identified using the Moth-Flame [25] and Ant Lion [26] heuristics. Global image descriptors are too coarse of a feature to predict pixel level properties. Mahomed and colleagues used Gaussian derivative filters to specify texture features on a per pixel level and used a support vector machine (SVM) for classification. To train their model, 858 pediatric chest X-ray images interpreted for PEP by three readers were used [2], which achieved a 0.850 auROC [12]. Furthermore, by specifying the features that are to be used by a computational prediction method, one may potentially omit features that can be directly learned from chest X-ray images, which will likely negatively impact model performance.

In light of the grading discrepancies found between readers interpreting pediatric chest X-ray images for pneumonia, our study will use multiple readers trained by the expert WHO X-ray image reading panel. Additionally, all readers will be blinded to the labels provided by the other readers. Furthermore, our study will use X-ray images collected from an LMIC hospital by radiologists for patient services. These images, collected over a period of 8 years, were not part of any specific radiological study and hence technologists taking the images were not trained in any specific manner, reflecting the variability commonly found in most hospitals. To demonstrate that our proposed system generalizes to other study sites, we will benchmark the system against images collected from two different sources: 1) the Kumudini Women's Medical College and Hospital (KWMCH) and 2) the WHO Chest Radiography in Epidemiological Studies (WHO CRES) working group [27].

For peer review only

Objectives

We aim to build a computational tool using a deep-learning approach to diagnose paediatric pneumonia using X-ray images assessed by multiple WHO-trained specialists. The primary project objectives are to:

(1) Collect and label ~10,000 paediatric chest X-rays images with high confidence, indicating if the right and left lung are healthy or if either lung meets the primary-end point pneumonia criteria

(2) Develop a software tool leveraging advances in the field of image recognition, in particular deep learning architectures and strategies for training these models, to diagnose paediatric pneumonia from chest X-ray images

(3) Evaluate the model against ~100 images collected from an alternative site labelled by the same readers of this study and against 130 independently collected images from sites differing from those described in this study a [27,28].

METHODS AND ANALYSIS

The overall design of the study is illustrated in Figure 1.

Participants and data source

Chest X-ray images will be derived from the ongoing WHO-coordinated Invasive Bacterial Vaccine Preventable Diseases (IB-VPD) Surveillance Platform in Bangladesh [29,30]. This surveillance system monitors burden of bacterial pneumonia, meningitis, and sepsis in children under 5 years old in-patients, using protocols described elsewhere [31]. Chest X-ray images are performed on the advice of the treating physicians Digital Imaging and Communications in Medicine (DICOM) images are collected by our team periodically. Technicians performing the chest X-rays are not trained for this specific project avoiding any bias in picture quality. Collection of the chest X-ray images started in 2013 and we expect to reach 10,000 chest X-ray images in 2021.

For peer review only

Two machines are used to capture the images: 1) SG Healthcare Jumong General and 2) TRIUP International Corp TRF100. The intent is for 100 images derived from the Kumudini Women's Medical College and Hospital (KWMCH), also an IB-VPD Surveillance Platform partner, to be included as a test set to assess the impact of images collected from different X-ray machines and technicians. The KWMCH site has two machines to capture images: 1) Hitachi Radnext 50 and 2) General Electric Dx-525. In the IB-VPD surveillance [31], written consent is obtained from parents or caregivers of all participants for other aspects of the study, including collection of data and additional analysis. Images of any participants that withdraw the consent from the surveillance study will not be considered for this study. The data generated from the individual patients will be deidentified prior to analysis.

Inclusion Criteria

An X-ray image will be included in this study if

- The age of the child for whom the X-ray is performed is <60 months,
- The X-ray was performed in one of the two study hospitals and the child was enrolled in the IB-VPD surveillance study, and
- The X-ray image captures the lung area

The inclusion criteria for the IB-VPD surveillance system for pneumonia are:

- The age of the child is <60 months,
- The child had coughing or difficulty breathing and tachypnea when calm at a rate of ≥60 breaths/min in an infant aged <2 months, or
- ≥50 breaths/min in an infant aged 2 to <12 months, or ≥40 breaths/min in children ≥12 months

For peer review only

Exclusion Criteria

Any image that is marked “uninterpretable” (features of the images are not interpretable with respect to presence or absence of primary end-point pneumonia) by the first two readers.

Sample size

In this study, we are attempting to train computational models rather than to assess if two populations differ. For this reason, we turn to literature to estimate the training dataset sizes required to build the proposed software tool to interpret paediatric chest X-rays. The tool to diagnose paediatric pneumonia (objective 2) will be composed of two stages: 1) lung detection to align the chest X-ray images to a reference image and 2) interpretation of paediatric chest X-rays for the presence of pneumonia.

To identify the location of lungs within the X-ray image, Dai and colleagues developed a generative adversarial network (GAN) and a baseline residual block network. They report Dice coefficient values of 97.3% and 96.3% respectively, where the Dice coefficient is a measure of similarity between the predicted location and a reference mask, with just 247 adult chest X-rays images [32]. The results will likely translate to the proposed study as we are interested in the identification of the same gross anatomical structures. This suggests that detecting the lung’s location within paediatric chest X-rays can readily be achieved with training using only a small proportion (about 300 from 10,000) of the collected chest X-ray images.

For interpretation of chest X rays, we will focus on studies that used expert panels to label X-ray images as a means to estimate the dataset size required to train a CNN to diagnose paediatric pneumonia directly from chest X-ray images. Prior studies used NLP systems to analyze radiology reports to programmatically assign labels, enabling large collections of chest X-ray images to be labelled [10,11]. However, due to the variability found between trained readers when diagnosing paediatric pneumonia from chest X-ray images [5,6], we believe that a more

For peer review only

appropriate strategy is to have each image assessed by multiple readers to ensure a high quality training dataset. Recent deep learning methods have diagnosed diseases such as tuberculosis after training on 1,007 chest X-ray images with a performance of 0.99 for the area under the receiver operator curve (auROC) [8] and diabetic retinopathy with a performance of 0.974 auROC using 128,175 retinal training images (28.1% were classified as referable diabetic retinopathy) [9]. For the diagnosis of referable diabetic retinopathy, Gulshan and colleagues found that using 10,000 retinal images to train their model resulted in a sensitivity and specificity respectively of 97% and 75% with saturation occurring at 60,000 images with a sensitivity and specificity respectively of 97.5% and 93.4%. We noted that the performance found between a reading panel and images labelled by an expert reader with a sensitivity and specificity respectively of 77% and 96.3% for paediatric pneumonia [6].

To date 8,002 paediatric chest X-ray images have been reviewed by a single clinician, of which 2,320 (29%) were identified with primary end-point pneumonia in either lung, 4786 (60%) had no pneumonia, and 898 (11%) were uninterpretable. We expect this class ratio to remain true for the complete dataset. Together, these studies and observations suggest that the 10,000 paediatric chest X-rays being collected will result in a suitable dataset to train a neural network to identify pneumonia cases among paediatric chest X-ray images.

Study Procedure

To build a software tool capable of diagnosing paediatric pneumonia directly from X-ray images, it is first necessary to establish a representative training dataset (Objective 1) that is of a sufficiently large size for CNN to be successfully trained (Figure 1). To this end, ~10,000 paediatric chest X-rays will be collected and labelled by radiologists/clinicians standardized to the WHO definitions (each blinded to all labels provided by the other readers) [4] who will interpret the images as having 1) primary end-point pneumonia (PEP), 2) other lung infiltrates, and/or 3) pleural fluid in either the left or right lung, for a total of six possible binary outcomes. Multiple labels for both lungs rather than a single binary value will enable information from

For peer review only

both lungs to be leveraged and information about other non-PEP classes may be used to guide the pneumonia prediction task. A challenge to assigning a pneumonia label to an image is the high discordance observed between diagnoses from trained radiologists / clinicians [5]. We plan on mitigating this by having multiple readers standardized to the WHO definitions to read each chest X-ray image. Specifically, two primary readers will assess all images meeting the described inclusion criteria. Chest X-ray images having discordant PEP labels will be reassessed by a third reader. A simple majority voting will be used to assign the multiclass labels for images. The remaining discordant labels (ie. not PEP) will be assigned randomly to reflect uncertainty in the label. Since "uninterpretable" images may still have evidence of some of the six outcomes, images labelled as "uninterpretable" by both readers will be excluded from the computational modeling process. Images labelled as "uninterpretable" by one primary reader but "interpretable" by the other will be re-evaluated by the third reader (Figure 1).

The software tool to diagnose paediatric pneumonia (objective 2) will be composed of two stages: 1) lung detection to align the chest X-ray images to a reference image and 2) interpretation of paediatric chest X-ray images for the presence of pneumonia. Chest X-ray images of paediatric patients can be highly variable, specifically in resource poor settings without well-trained personnel and/or high patient flow, ranging from images of uncentered whole child bodies to properly aligned lung images (Figure 2). To aid the paediatric pneumonia prediction task, we first aim to identify the location of the lung. While techniques such as data augmentation can minimize the impact of translation, scaling, and rotation on a neural network's performance, explicitly removing these sources of variation can improve performance [33]. We will evaluate two approaches for their ability to identify the lungs location within an X-ray image. The first model's architecture is a variant of the VGG (Visual Geometry Group) architecture, but with residual network blocks for pixel-level object detection. This has previously been shown to work well for adult lung segmentation [32,34], in combination with image histogram equalization. This architecture of this lung detection model will be determined from a hyper-parameter search, which will match the model's complexity to the lung detection problem. The lung detection model will initially be trained on 300 paediatric

For peer review only

chest X-ray images and evaluated on a validation set of an additional 100 paediatric chest X-ray images and assessed using the Dice coefficient to evaluate the model's performance. Additional images may be used if the model's performance is deemed insufficient. In line with observations by Dai and colleagues [32], 300 images should be sufficient for our lung segmentation and centering task. Key point detection on the segmented images by Binary Robust Invariant Scalable Keypoints (BRISK) [35] or Fast Retina Keypoint (FREAK) [36] to align the images to a reference image using the Open Source Computer Vision Library (OpenCV), an image manipulation toolkit [37]. The second approach for lung detection will be to explore the use of spatial transformer networks [33] in conjunction with object recognition deep learning architectures, thereby enabling a single model to detect the lung and diagnose paediatric pneumonia.

For peer review only

The second stage of the software tool will diagnose pneumonia from the aligned paediatric chest X-ray images. This identification task will be performed in a multi-task prediction setting, namely that the CNN's goal will be to predict 1) primary end-point pneumonia, 2) other lung infiltrates, and/or 3) pleural fluid in both the left and right lung for a total of 6 binary labels. The pneumonia diagnosis model will be based on the DenseNet architecture [18]. Dilated convolutions [38] will be incorporated into the architecture to aggregate features spanning different distances across the lungs. The final model architecture will be determined from an architecture hyper parameter search over the number of convolution layers per dense block, feature-map growth rate, and feature-map compression will be explored. The number of dense blocks will be kept fixed to four. The binary cross entropy loss function will be used for model training, to assess differences between the model's prediction and the ground truth provided by the labels and to guide how the model parameters are to be updated to improve the model predictions. An ensemble of five models will form the final predictor, whose predictions for each of the 6 output classes will be the unweighted average of the five models' predicted class probabilities.

Statistical Methods and Performance Evaluation

To evaluate the quality of the labels associated with the ~10,000 chest X-ray images, we will evaluate the readers' concordance with each other and with the compiled multi-reader labels using Cohen's kappa coefficient, sensitivity, and specificity for both the right and left lung. 95% confidence intervals will be assessed empirically by bootstrapping.

To select the model hyper-parameters (model complexity), the data will be partitioned into 5-folds. This will allow us to train models on 80% and validate on 20% of the data and to compare models with different hyper-parameters. Given the prevalence of pneumonia within this dataset measured to date (29%), having enough positive samples to validate the model for both the right and left lung is a concern, which we believe a 5-fold cross validation addresses. Hyper-parameters will be determined with a mixture of grid searching and prior literature values. These five models will form an ensemble and the final predictions for each of the 6 output classes will be formed by taking the unweighted average of the predicted class probabilities for each label.

Two image datasets (objective 3) will be used to evaluate the software tool. The first test set will be from KWMCH, whose labels will be provided by the same readers who labelled the training image dataset. This dataset will enable the study to evaluate the impact of using different X-ray imagining machines and technicians and disease prevalence. The second dataset will be from WHO CRES, whose images were independently collected and independently labelled by clinicians and radiologists following the WHO Radiological Pneumonia definitions [4]. This will assess how transferable our method will be to other study sites. Performance will be evaluated using the area under the receiver operator curve (auROC), the area under the precision-recall curve (auPR), sensitivity, and specificity. Finally, 95% confidence intervals will be assessed empirically by bootstrapping.

For peer review only

Patient and Public Involvement

No patient or members of the public were involved in this study.

DISCUSSION

The study will be the first to use a large dataset whose labels are derived from multiple expert readers to train a deep-learning model to diagnose paediatric primary-end point pneumonia. Prior work to identify pneumonia in adult lungs using a CNN achieved a performance of 0.931 auROC and a 0.815 auROC on an independently collected benchmark. This computational model was trained on chest X-ray images labelled from a single reader whose pneumonia labels were extracted from radiology reports via a NLP system that may introduce extraction errors [11]. A prior study that leverage multiple expert readers, was small in size, containing 858 pediatric chest X-ray images interpreted for PEP by three readers [2]. Since the X-rays were collected over a short period of time, this dataset likely lacks the image variance observed in LMIC settings. The study authors used a SVM to make pixel level predictions and a 95th percentile pixel score as an image score to achieve a 0.850 auROC [12]. We are expecting our model to have similar or better performance since the training data is evaluated by multiple readers and the goal of diagnosing primary-end point pneumonia is better defined.

A limitation of the study is the use of chest X-ray images from individuals ≤ 60 months of age derived from a single institution using two machines to capture the images to construct a computational model to interpret chest X-ray images for the presence of pneumonia. Findings from the study may not generalize to individuals ≥60 months of age. However, this is the most appropriate age group for this study as more than 90% of pediatric pneumonia cases are limited to children <5 years of age.

While the computational model will be evaluated against an independently collected and labelled chest X-ray image dataset for the presence or absence of primary end-point

For peer review only

pneumonia (WHO CRES dataset), some findings may not generalize to other settings (as the training data is from a single institution). To overcome this limitation, we are aiming to extend this study as a multi-country surveillance, if successful.

Data Availability

All findings will be published in an open-access journal. All X-ray labels and statistical code will be made openly available. The model and images will be made available upon request.

Acknowledgements

This research was commissioned by the UK National Institute for Health Research (NIHR) Global Health Research Unit on Respiratory Health (RESPIRE), using UK Aid from the UK Government. The views expressed in this publication are those of the author(s) and not necessarily those of the NIHR or the UK Department of Health and Social Care. We are grateful to Mohammad Shahidul Islam and Md. Shariful Islam for the intellectual and technical guidance.

Author’s Contributors

Conceptual construction of research and design: MGFS, SS, and SKS. Critical manuscript review: MGFS, SS, SKS, SAS, SL and HN. All authors have approved the final version of the manuscript for submission for publication.

Funding

This work has received funding support from NIHR Global Health Research Unit on Respiratory Health (RESPIRE) 16/136/109.

For peer review only

Competing interests

None declared

Ethics

All study protocols were approved by the Ethical Review Committees of the Bangladesh Institute of Child Health.

References

1 McAllister DA, Liu L, Shi T, *et al.* Global, regional, and national estimates of pneumonia morbidity and mortality in children younger than 5 years between 2000 and 2015: a systematic analysis. *Lancet Glob Health* 2019;**7**:e47–57.

2 O'Brien KL, Baggett HC, Brooks WA, *et al.* Causes of severe pneumonia requiring hospital admission in children without HIV infection from Africa and Asia: the PERCH multi-country case-control study. *The Lancet* 2019.

3 National Institute of Population Research and Training. BANGLADESH DEMOGRAPHIC AND HEALTH SURVEY. 2014.https://dhsprogram.com/pubs/pdf/FR311/FR311.pdf

4 Cherian T, Mulholland EK, Carlin JB, *et al.* Standardized interpretation of paediatric chest radiographs for the diagnosis of pneumonia in epidemiological studies. *Bull World Health Organ* 2005;**83**:353–9.

5 Elemraid MA, Muller M, Spencer DA, *et al.* Accuracy of the interpretation of chest radiographs for the diagnosis of paediatric pneumonia. *PLoS One* 2014;**9**:e106051.

6 McCollum ED, Ahmed S, Chowdhury NH, *et al.* Chest radiograph reading panel performance in a Bangladesh pneumococcal vaccine effectiveness study. *BMJ Open Respir Res* 2019;**6**:e000393.

7 Liu Y, Gadepalli K, Norouzi M, *et al.* Detecting cancer metastases on gigapixel pathology images. *ArXiv Prepr ArXiv170302442* 2017.

8 Lakhani P, Sundaram B. Deep learning at chest radiography: automated classification of pulmonary tuberculosis by using convolutional neural networks. *Radiology* 2017;**284**:574–82.

9 Gulshan V, Peng L, Coram M, *et al.* Development and validation of a deep learning algorithm for detection of diabetic retinopathy in retinal fundus photographs. *Jama* 2016;**316**:2402–10.

For peer review only

445 10 Rajpurkar P, Irvin J, Zhu K, *et al.* Chexnet: Radiologist-level pneumonia detection on
446 chest x-rays with deep learning. *ArXiv Prepr ArXiv171105225* 2017.

447 11 Zech JR, Badgeley MA, Liu M, *et al.* Variable generalization performance of a deep
448 learning model to detect pneumonia in chest radiographs: A cross-sectional study. *PLoS Med*
449 2018;**15**:e1002683.

450 12 Mahomed N, van Ginneken B, Philipsen RHHM, *et al.* Computer-aided diagnosis for
451 World Health Organization-defined chest radiograph primary-endpoint pneumonia in
452 children. *Pediatr Radiol* 2020;**50**:482–91. doi:10.1007/s00247-019-04593-0

453 13 Madhi SA, Klugman KP. World Health Organisation definition of "radiologically-
454 confirmed pneumonia" may under-estimate the true public health value of conjugate
455 pneumococcal vaccines. *Vaccine* 2007;**25**:2413–9. doi:10.1016/j.vaccine.2006.09.010

456 14 Russakovsky O, Deng J, Su H, *et al.* Imagenet large scale visual recognition challenge. *Int*
457 *J Comput Vis* 2015;**115**:211–52.

458 15 Shin H-C, Roth HR, Gao M, *et al.* Deep convolutional neural networks for computer-
459 aided detection: CNN architectures, dataset characteristics and transfer learning. *IEEE Trans*
460 *Med Imaging* 2016;**35**:1285–98.

461 16 Chouhan V, Singh SK, Khamparia A, *et al.* A novel transfer learning based approach for
462 pneumonia detection in chest X-ray images. *Appl Sci* 2020;**10**:559.

463 17 Krizhevsky A, Sutskever I, Hinton GE. Imagenet classification with deep convolutional
464 neural networks. *Commun ACM* 2017;**60**:84–90.

465 18 Huang G, Liu Z, Van Der Maaten L, *et al.* Densely connected convolutional networks. In:
466 *Proceedings of the IEEE conference on computer vision and pattern recognition*. 2017. 4700–
467 8.

468 19 Szegedy C, Vanhoucke V, Ioffe S, *et al.* Rethinking the inception architecture for
469 computer vision. In: *Proceedings of the IEEE conference on computer vision and pattern*
470 *recognition*. 2016. 2818–26.

471 20 Szegedy C, Liu W, Jia Y, *et al.* Going deeper with convolutions. In: *Proceedings of the IEEE*
472 *conference on computer vision and pattern recognition*. 2015. 1–9.

473 21 He K, Zhang X, Ren S, *et al.* Deep residual learning for image recognition. In: *Proceedings*
474 *of the IEEE conference on computer vision and pattern recognition*. 2016. 770–8.

475 22 Kermany DS, Goldbaum M, Cai W, *et al.* Identifying medical diagnoses and treatable
476 diseases by image-based deep learning. *Cell* 2018;**172**:1122–31.

For peer review only

23 Hashmi MF, Katiyar S, Keskar AG, *et al.* Efficient pneumonia detection in chest xray images using deep transfer learning. *Diagnostics* 2020;**10**:417.

24 Ke Q, Zhang J, Wei W, *et al.* A neuro-heuristic approach for recognition of lung diseases from X-ray images.

25 Mirjalili S. Moth-flame optimization algorithm: A novel nature-inspired heuristic paradigm. *Knowl-Based Syst* 2015;**89**:228–49.

26 Mirjalili S. The ant lion optimizer. *Adv Eng Softw* 2015;**83**:80–98.

27 WHO Chest Radiography in Epidemiological Studies (CRES). https://who-cres.mcri.edu.au/

28 Mahomed N, Fancourt N, De Campo J, *et al.* Preliminary report from the World Health Organisation chest radiography in epidemiological studies project. *Pediatr Radiol* 2017;**47**:1399–404.

29 Hasan AZ, Saha S, Saha SK, *et al.* Using pneumococcal and rotavirus surveillance in vaccine decision-making: A series of case studies in Bangladesh, Armenia and the Gambia. *Vaccine* 2018;**36**:4939–43. doi:10.1016/j.vaccine.2018.06.001

30 Global Invasive Bacterial Vaccine-Preventable Diseases Surveillance — 2008–2014. https://www.cdc.gov/mmwr/preview/mmwrhtml/mm6349a4.htm (accessed 2 Sep 2020).

31 Saha SK, Hossain B, Islam M, *et al.* Epidemiology of invasive pneumococcal disease in Bangladeshi children before introduction of pneumococcal conjugate vaccine. *Pediatr Infect Dis J* 2016;**35**:655–61.

32 Dai W, Dong N, Wang Z, *et al.* Scan: Structure correcting adversarial network for organ segmentation in chest x-rays. In: *Deep Learning in Medical Image Analysis and Multimodal Learning for Clinical Decision Support*. Springer 2018. 263–73.

33 Jaderberg M, Simonyan K, Zisserman A, *et al.* Spatial transformer networks. In: *Advances in neural information processing systems*. 2015. 2017–25.

34 Bansal A, Chen X, Russell B, *et al.* Pixelnet: Towards a general pixel-level architecture. *ArXiv Prepr ArXiv160906694* 2016.

35 Leutenegger S, Chli M, Siegwart RY. BRISK: Binary robust invariant scalable keypoints. In: *2011 International conference on computer vision*. Ieee 2011. 2548–55.

36 Alahi A, Ortiz R, Vandergheynst P. Freak: Fast retina keypoint. In: *2012 IEEE Conference on Computer Vision and Pattern Recognition*. Ieee 2012. 510–7.

37 Bradski G. The OpenCV Library. *Dr Dobbs J Softw Tools* 2000.

For peer review only

38 Yu F, Koltun V. Multi-scale context aggregation by dilated convolutions. *ArXiv Prepr ArXiv151107122* 2015.

FIGURE LEGENDS

Figure 1: Study design schema. PEP: Primary end point pneumonia; KWMCH : Kumudini Women's Medical College Hospital

Figure 2: Digitized chest X-ray images include (Left) full body images and (Right) images of just the lung area.

For peer review only

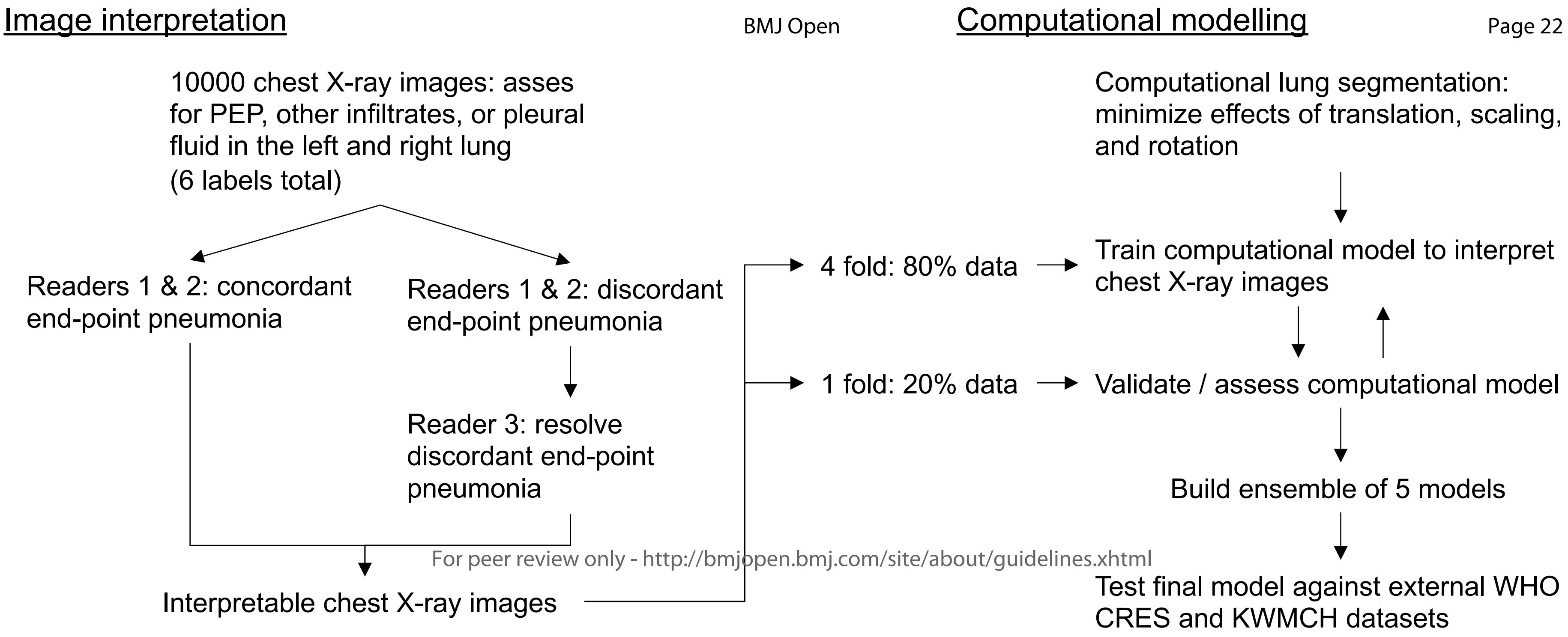

For peer review only - http://bmjopen.bmj.com/site/about/guidelines.xhtml

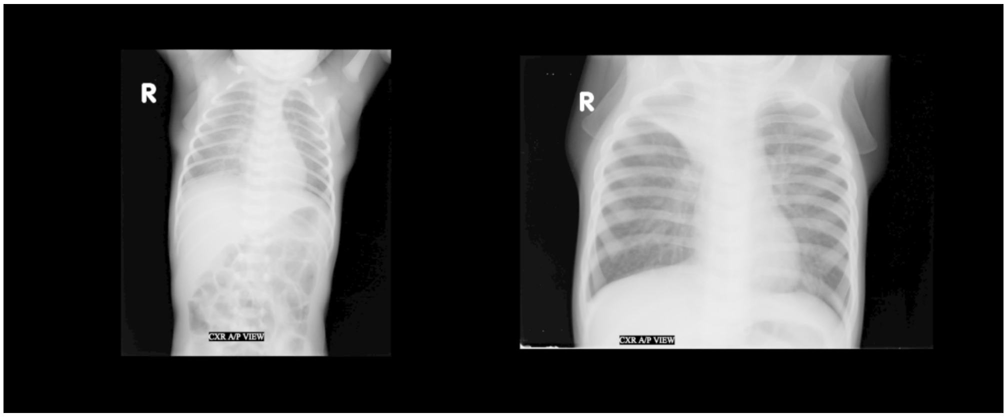

Figure 2: Digitized chest X-ray images include (Left) full body images and (Right) images of just the lung area.

BMJ Open

A study protocol and design for the assessment of paediatric pneumonia from X-Ray images using deep learning

Journal:	*BMJ Open*
Manuscript ID	bmjopen-2020-044461.R2
Article Type:	Protocol
Date Submitted by the Author:	20-Feb-2021

Complete List of Authors:	Sun, Mark; Child Health Research Foundation<br>Saha, Senjuti; Child Health Research Foundation; Johns Hopkins University Bloomberg School of Public Health,<br>Shah, Syed Ahmar; The University of Edinburgh Usher Institute of Population Health Sciences and Informatics,<br>Luz, Saturnino; University of Edinburgh School of Molecular Genetic and Population Health Sciences, Usher Institute of Population Health Sciences and Informatics<br>Nair, Harish; University of Edinburgh School of Molecular Genetic and Population Health Sciences,<br>Saha, Samir; Child Health Research Foundation

**Primary Subject Heading**:	Diagnostics
Secondary Subject Heading:	Diagnostics, Health informatics, Infectious diseases, Public health, Respiratory medicine
Keywords:	Paediatric radiology < PAEDIATRICS, Respiratory infections < THORACIC MEDICINE, Public health < INFECTIOUS DISEASES, Chest imaging < RADIOLOGY & IMAGING, Diagnostic radiology < RADIOLOGY & IMAGING

SCHOLARONE™
Manuscripts

I, the Submitting Author has the right to grant and does grant on behalf of all authors of the Work (as defined in the below author licence), an exclusive licence and/or a non-exclusive licence for contributions from authors who are: i) UK Crown employees; ii) where BMJ has agreed a CC-BY licence shall apply, and/or iii) in accordance with the terms applicable for US Federal Government officers or employees acting as part of their official duties; on a worldwide, perpetual, irrevocable, royalty-free basis to BMJ Publishing Group Ltd ("BMJ") its licensees and where the relevant Journal is co-owned by BMJ to the co-owners of the Journal, to publish the Work in this journal and any other BMJ products and to exploit all rights, as set out in our licence.

The Submitting Author accepts and understands that any supply made under these terms is made by BMJ to the Submitting Author unless you are acting as an employee on behalf of your employer or a postgraduate student of an affiliated institution which is paying any applicable article publishing charge ("APC") for Open Access articles. Where the Submitting Author wishes to make the Work available on an Open Access basis (and intends to pay the relevant APC), the terms of reuse of such Open Access shall be governed by a Creative Commons licence – details of these licences and which Creative Commons licence will apply to this Work are set out in our licence referred to above.

Other than as permitted in any relevant BMJ Author's Self Archiving Policies, I confirm this Work has not been accepted for publication elsewhere, is not being considered for publication elsewhere and does not duplicate material already published. I confirm all authors consent to publication of this Work and authorise the granting of this licence.

For peer review only

A study protocol and design for the assessment of paediatric pneumonia from X-Ray images using deep learning

Mark George Ford Sun*[1], Senjuti Saha*[1,2,#], Syed Ahmar Shah[5], Saturnino Luz[3,5], Harish Nair[4,5], Samir K Saha[1,4,#]

[1]Child Health Research Foundation, Dhaka, Bangladesh

[2]Johns Hopkins Bloomberg School of Public Health, USA

[3]Centre for Global Health, Usher Institute, University of Edinburgh

[4]Dhaka Shishu (Children) Hospital, Bangladesh

[5]NIHR Global Health Research Unit on Respiratory Health (RESPIRE), Usher Institute, University of Edinburgh, Edinburgh, UK

*These authors made equal contribution to the paper

#Co-correspondence to:

Prof Samir K Saha, PhD, FRCPath
Dhaka Shishu Hospital & Bangladesh Institute of Child Health
Child Health Research Foundation
23/2 Sel Huq Skypark, Block-B Khilji Road,
Dhaka 1207, Bangladesh
samir@chrfbd.org; +8801713461254

Senjuti Saha, PhD
Child Health Research Foundation
23/2 Sel Huq Skypark, Block-B Khilji Road,
Dhaka 1207, Bangladesh
senjutisaha@chrfbd.org; +88 01711646038

Word count: 300 (abstract); 3,248 (main text)

For peer review only

ABSTRACT

Introduction

In low and middle-income countries, pneumonia remains the leading cause of illness and death in children <5 years. The recommended tool for diagnosing paediatric pneumonia is the interpretation of chest X-ray images, which is difficult to standardize and requires trained clinicians/radiologists. Current automated computational tools have primarily focused on assessing adult pneumonia and were trained on images evaluated by a single specialist. We aim to provide a computational tool using a deep-learning approach to diagnose paediatric pneumonia using X-ray images assessed by multiple specialists trained by the WHO expert X-ray image reading panel.

Methods and analysis

Approximately 10,000 paediatric chest X-ray images are currently being collected from an ongoing WHO-supported surveillance study in Bangladesh. Each image will be read by two trained clinicians/radiologists for the presence or absence of primary end-point pneumonia (PEP) in each lung, as defined by the WHO. Images whose PEP labels are discordant in either lung will be reviewed by a third specialist and the final assignment will be made using a majority vote. Convolutional neural networks will be used for lung segmentation to align and scale the images to a reference, and for interpretation of the images for the presence of PEP. The model will be evaluated against an independently collected and labelled set of images from the WHO. The study outcome will be an automated method for the interpretation of chest radiographs for diagnosing pediatric pneumonia.

Ethics and Dissemination

All study protocols were approved by the Ethical Review Committees of the Bangladesh Institute of Child Health, Bangladesh. The study sponsor deemed it unnecessary to attain ethical approval from the Academic and Clinical Central Office for Research and Development of University of Edinburgh, UK. The study uses existing X-ray images from an ongoing WHO-coordinated surveillance. All findings will be published in an open-access journal. All X-ray labels

For peer review only

For peer review only - http://bmjopen.bmj.com/site/about/guidelines.xhtml

and statistical code will be made openly available. The model and images will be made available upon request.

Strengths and limitations of this study

- The main strength of the study is that it will employ a large, high confidence dataset of ~10,000 paediatric chest X-ray images interpreted by multiple trained readers for the presence of primary endpoint pneumonia.
- Another strength is that the computational tool will be evaluated against two external paediatric chest X-ray image datasets to assess generalizability of the model.
- The primary limitation of the study is that the computational model will be trained from chest X-ray images derived from a single study site.

For peer review only

INTRODUCTION

Paediatric pneumonia overview

Pneumonia is a leading cause of childhood illness and death in developing countries. Globally, about 138 million cases and 0.9 million deaths are estimated to occur annually due to pneumonia with about six million cases in Bangladesh alone [1]. However, determining the disease prevalence is complicated due to the lack of reliable diagnostic tools: 1) blood cultures are insensitive as most cases are likely not bacteremic and therefore a blood culture cannot determine a bacterial respiratory infection and 2) other diagnostic procedures like radiological findings are difficult to standardize. While the true proportions of pneumonia etiologies (viral/bacterial) are unknown [2], 34% of children in Bangladesh diagnosed with pneumonia receive empirical antibiotics [3]. Image based diagnostics such as the interpretation of chest X-ray images to identify paediatric pneumonia are regarded as the "gold standard" [4], but they are difficult to standardize and the interpretations can vary between radiologists and clinicians [5,6]. Furthermore, the limited number of well-trained radiologists and clinicians limits the use of X-ray images as a ubiquitous diagnostic tool for pneumonia. This lack of accurate diagnosis hampers the determination of the true burden of pneumonia, and the proportion that is preventable by vaccination, which in turn impedes institution of evidence-based intervention strategies and vaccine impact measurements.

Deep learning methods such as convolutional neural networks (CNNs) may be used to expedite the adoption of chest X-ray images for the diagnosis of pneumonia. CNNs are composed of multiple feature extractors stacked on top of each other. During the training process the feature extractors near the model input learn primitive image features such as edges, whereas feature extractors deeper into the model learn domain specific relationships important for the diagnostic task. CNNs have successfully been applied to assist in the detection and classification of human diseases like breast cancer [7,8], tuberculosis [9], diabetic retinopathy [10] and pneumonia [11–13] directly from images. In some instances, these systems performed as well as trained health professionals. While systems have been developed to identify paediatric

For peer review only

pneumonia from X-ray images, the datasets used for training are either small (<1000), evaluated on X-ray images derived from the same study site, or don't compare the model's performance with respect to a clinician/radiologist. Our goal is to construct a computational framework to automatically and systematically interpret paediatric chest X-ray images to diagnose pneumonia using ~10 000 training images and whose performance both generalized across datasets and is benchmarked to that of a clinician / radiologist. If successful, an automated system to detect pediatric pneumonia will have significant public health benefits. It will accelerate the adoption of chest X-ray images in low- and middle-income country (LMIC) settings, where there is a lack of trained radiologists and clinicians, and contribute to the standardization of X-ray image analysis. This will encourage clinicians to rationalise the use of antibiotic therapies and to facilitate antibiotic stewardship.

Diagnosing paediatric pneumonia

Chest X-ray images interpreted to have primary endpoint pneumonia (PEP) are considered the "gold standard" for the diagnosis of paediatric pneumonia [4]. The PEP case definition was developed by the WHO as part of clinical trials to assess the efficacy of vaccines against *Streptococcus pneumoniae* and *Haemophilus influenza* type b (Hib) and is defined as the presence of end-point consolidation or pleural effusion [4]. Despite efforts to standardize the interpretation of chest radiographs by training specialists to the WHO definitions, significant discrepancies are still observed between readers interpreting the same chest X-ray images, due to the subjective nature of the diagnosis [5,6]. To overcome differences between trained readers, a panel of multiple readers may be employed to interpret images [6].

Establishing well-funded and rigorous clinical trials with multiple trained readers to evaluate every chest X-ray image is an onerous task. This time intensive task may be alleviated with the aid of computational tools to diagnose pneumonia directly from chest X-ray images. CNN models are one such tool, but require thousands of labelled images to successfully be trained from a randomly initialized state. To this end, adult radiology reports have been analyzed by

For peer review only

natural language processing (NLP) systems to programmatically assign labels. This enabled the collection of a large computationally labelled chest X-ray images (40,000 – 110,000 images) for the training of CNN models to diagnose pneumonia directly from adult chest X-ray images [11,12,14]. Transfer learning can be applied to reduce model training time or to enable model training should the dataset be too small to train a model from a randomly initialized state. Models are first pretrained on a large image dataset such as ImageNet [15], containing more than a million labelled images of common entities. These pretrained models are transferred to the domain of interest by finetuning the model [16]. Zech and colleagues found that transfer learning for a large adult chest X-ray image dataset achieved a performance of 0.931 for the area under the receiver operator curve (auROC). When evaluated on an external benchmark dataset, whose data source differs from that of the training dataset, the model performed significantly worse with an auROC of 0.815. This performance degradation of models trained on a single site was found in 3 out of 5 instances [12], highlighting the need of external evaluation datasets to avoid the reporting of inflated performance metrics, which likely holds true in the paediatric pneumonia setting.

Model training from randomly initialize weights [17,18] and model finetuning has been applied for the diagnosis of paediatric pneumonia directly from X-ray images [19–21]. Efforts to increase performance within the paediatric pneumonia setting have involved using multiple different architectures to form model ensembles, thereby expanding the space of functions that can be learned to associate an X-ray image to a diagnosis label [22,23]. Chouhan and colleagues used transfer learning with five different models [22] based on the AlexNet [24], DenseNet-121 [25], Inception V3 [26], GoogLeNet [27], and ResNet18 [28] architectures, for a pediatric pneumonia X-ray image dataset containing 5856 labelled images [19]. Combining the individual model predictions by a majority vote (99.34 auROC) [22] or in a linear combination scheme (99.76 auROC) [23] were suggestive that a performance increase relative to a single model is possible. Rather than combining the final scalar prediction scores, multiple CNN models may be treated as feature extractors that may be used as input to an attention mechanism, enabling the model to attend to import features. Cha and colleagues used the Self-

Attention mechanism [29] over the ensemble feature vectors to achieve an auROC of 96.03 [30]. Feature selection may be applied over the ensemble feature vector using the minimum redundancy maximum relevance (mRMR) method. The selected features may be used as input into a classifier such as a support vector machine (SVM) [31]. Other feature selection schemes include the use of Artificial Ecosystem-based Optimization (AEO) [32,33] and the Marine Predators Algorithm (MPA) enhanced by fractional-order calculus (FO) [34] , which have respectively been applied for the diagnosis of tuberculosis and COVID-19 from X-ray images. Alternatively, the prediction of a CNN may be gated by an anomaly detection scheme using a modified loss function to guide model training [35]. These methods were evaluated on images from the same study site, thus additional investigation is required to ensure that the models are learning discriminatory radiological features of pneumonia and are not exploiting confounding features of the study site.

During CNN model training, image features responsible for the radiological diagnosis of pneumonia are learned. Rather than learning the image features directly from the training dataset, images features may be explicitly specified. Ke and colleagues used global image descriptors of hue, saturation, and brightness as input to a fully connected neural network to predict pneumonia [36]. Representative pixels of disease lung tissue were identified using the Moth-Flame [37] and Ant Lion [38] heuristics. Global image descriptors are too coarse of a feature to predict pixel level properties. Mahomed and colleagues used Gaussian derivative filters to specify texture features on a per pixel level and used a support vector machine (SVM) for classification. A small pediatric dataset of 858 chest X-ray images was interpreted for PEP by three readers [2] was used to train there model, which achieved a 0.850 auROC [13]. Our study will use 10,000 images interpreted for PEP. Furthermore, by specifying the features that are to be used by a computational prediction method, one may potentially omit features that can be directly learned from chest X-ray images, which will likely negatively impact model performance.

For peer review only

In light of the grading discrepancies found between readers interpreting pediatric chest X-ray images for pneumonia, our study will use multiple readers trained by the expert WHO X-ray image reading panel. Additionally, all readers will be blinded to the labels provided by the other readers. Furthermore, our study will use X-ray images collected from an LMIC hospital by radiologists for patient services. These images, collected over a period of 8 years, were not part of any specific radiological study. Hence, technologists taking the images were not uniformly trained specifically for this study, reflecting the variability commonly found in most hospitals. To demonstrate that our proposed system generalizes to other study sites, we will benchmark the system against images collected from two different sources: 1) the Kumudini Women's Medical College and Hospital (KWMCH) and 2) the WHO Chest Radiography in Epidemiological Studies (WHO CRES) working group [39].

Objectives

We aim to build a computational tool using a deep-learning approach to diagnose paediatric pneumonia using X-ray images assessed by multiple WHO-trained specialists. The primary project objectives are to:

(1) Collect and label ~10,000 paediatric chest X-rays images with high confidence, indicating if the right and left lung are healthy or if either lung meets the primary-end point pneumonia criteria
(2) Develop a software tool leveraging advances in the field of image recognition, in particular deep learning architectures and strategies for training these models, to diagnose paediatric pneumonia from chest X-ray images
(3) Evaluate the model against ~100 images collected from an alternative site labelled by the same readers of this study and against 130 independently collected images from sites differing from those described in this study a [39,40].

For peer review only

METHODS AND ANALYSIS

The overall design of the study is illustrated in Figure 1.

Participants and data source

Chest X-ray images will be derived from the ongoing WHO-coordinated Invasive Bacterial Vaccine Preventable Diseases (IB-VPD) Surveillance Platform in Bangladesh [41,42]. This surveillance system monitors burden of bacterial pneumonia, meningitis, and sepsis in children under 5 years old in-patients, using protocols described elsewhere [43]. Chest X-ray images are performed on the advice of the treating physicians Digital Imaging and Communications in Medicine (DICOM) images are collected by our team periodically. Technicians performing the chest X-rays are not trained for this specific project avoiding any bias in picture quality. Collection of the chest X-ray images started in 2013 and we expect to reach 10,000 chest X-ray images in 2021.

Two machines are used to capture the images: 1) SG Healthcare Jumong General and 2) TRIUP International Corp TRF100. The intent is for 100 images derived from the Kumudini Women's Medical College and Hospital (KWMCH), also an IB-VPD Surveillance Platform partner, to be included as a test set to assess the impact of images collected from different X-ray machines and technicians. The KWMCH site has two machines to capture images: 1) Hitachi Radnext 50 and 2) General Electric Dx-525. In the IB-VPD surveillance , written consent is obtained from parents or caregivers of all participants for other aspects of the study, including collection of data and additional analysis. Images of any participants that withdraw the consent from the surveillance study will not be considered for this study. The data generated from the individual patients will be deidentified prior to analysis.

For peer review only

Inclusion Criteria

An X-ray image will be included in this study if

- The age of the child for whom the X-ray is performed is <60 months,
- The X-ray was performed in one of the two study hospitals and the child was enrolled in the IB-VPD surveillance study, and
- The X-ray image captures the lung area

The inclusion criteria for the IB-VPD surveillance system for pneumonia are:

- The age of the child is <60 months,
- The child had coughing or difficulty breathing and tachypnea when calm at a rate of ≥60 breaths/min in an infant aged <2 months, or
- ≥50 breaths/min in an infant aged 2 to <12 months, or ≥40 breaths/min in children ≥12 months

Exclusion Criteria

Any image that is marked "uninterpretable" (features of the images are not interpretable with respect to presence or absence of primary end-point pneumonia) by the first two readers.

Sample size

In this study, we are attempting to train computational models rather than to assess if two populations differ. For this reason, we turn to literature to estimate the training dataset sizes required to build the proposed software tool to interpret paediatric chest X-rays. The tool to diagnose paediatric pneumonia (objective 2) will be composed of two stages: 1) lung detection to align the chest X-ray images to a reference image and 2) interpretation of paediatric chest X-rays for the presence of pneumonia.

For peer review only

To identify the location of lungs within the X-ray image, Dai and colleagues developed a generative adversarial network (GAN) and a baseline residual block network. They report Dice coefficient values of 97.3% and 96.3% respectively, where the Dice coefficient is a measure of similarity between the predicted location and a reference mask, with just 247 adult chest X-rays images [44]. The results will likely translate to the proposed study as we are interested in the identification of the same gross anatomical structures. This suggests that detecting the lung's location within paediatric chest X-rays can readily be achieved with training using only a small proportion (about 300 from 10,000) of the collected chest X-ray images.

For interpretation of chest X rays, we will focus on studies that used expert panels to label X-ray images as a means to estimate the dataset size required to train a CNN to diagnose paediatric pneumonia directly from chest X-ray images. Prior studies used NLP systems to analyze radiology reports to programmatically assign labels, enabling large collections of chest X-ray images to be labelled [11,12]. However, due to the variability found between trained readers when diagnosing paediatric pneumonia from chest X-ray images [5,6], we believe that a more appropriate strategy is to have each image assessed by multiple readers to ensure a high quality training dataset. Recent deep learning methods have diagnosed diseases such as tuberculosis after training on 1,007 chest X-ray images with a performance of 0.99 for the area under the receiver operator curve (auROC) [9] and diabetic retinopathy with a performance of 0.974 auROC using 128,175 retinal training images (28.1% were classified as referable diabetic retinopathy) [10]. For the diagnosis of referable diabetic retinopathy, Gulshan and colleagues found that using 10,000 retinal images to train their model resulted in a sensitivity and specificity respectively of 97% and 75% with saturation occurring at 60,000 images with a sensitivity and specificity respectively of 97.5% and 93.4%. We noted that the performance found between a reading panel and images labelled by an expert reader with a sensitivity and specificity respectively of 77% and 96.3% for paediatric pneumonia [6].

For peer review only

283 To date 8,002 paediatric chest X-ray images have been reviewed by a single clinician, of which 2,320 (29%) were identified with primary end-point pneumonia in either lung, 4786 (60%) had no pneumonia, and 898 (11%) were uninterpretable. We expect this class ratio to remain true for the complete dataset. Together, these studies and observations suggest that the 10,000 paediatric chest X-rays being collected will result in a suitable dataset to train a neural network to identify pneumonia cases among paediatric chest X-ray images.

Study Procedure

To build a software tool capable of diagnosing paediatric pneumonia directly from X-ray images, it is first necessary to establish a representative training dataset (Objective 1) that is of a sufficiently large size for CNN to be successfully trained (Figure 1). To this end, ~10,000 paediatric chest X-rays will be collected and labelled by radiologists/clinicians standardized to the WHO definitions (each blinded to all labels provided by the other readers) [4] who will interpret the images as having 1) primary end-point pneumonia (PEP), 2) other lung infiltrates, and/or 3) pleural fluid in either the left or right lung, for a total of six possible binary outcomes. Multiple labels for both lungs rather than a single binary value will enable information from both lungs to be leveraged and information about other non-PEP classes may be used to guide the pneumonia prediction task. A challenge to assigning a pneumonia label to an image is the high discordance observed between diagnoses from trained radiologists / clinicians [5]. We plan on mitigating this by having multiple readers standardized to the WHO definitions to read each chest X-ray image. Specifically, two primary readers will assess all images meeting the described inclusion criteria. Chest X-ray images having discordant PEP labels will be reassessed by a third reader. A simple majority voting will be used to assign the multiclass labels for images. The remaining discordant labels (ie. not PEP) will be assigned randomly to reflect uncertainty in the label. Since “uninterpretable” images may still have evidence of some of the six outcomes, images labelled as “uninterpretable” by both readers will be excluded from the computational modeling process. Images labelled as “uninterpretable” by one primary reader but “interpretable” by the other will be re-evaluated by the third reader (Figure 1).

For peer review only

The software tool to diagnose paediatric pneumonia (objective 2) will be composed of two stages: 1) lung detection to align the chest X-ray images to a reference image and 2) interpretation of paediatric chest X-ray images for the presence of pneumonia. Chest X-ray images of paediatric patients can be highly variable, specifically in resource poor settings without well-trained personnel and/or high patient flow, ranging from images of uncentered whole child bodies to properly aligned lung images (Figure 2). To aid the paediatric pneumonia prediction task, we first aim to identify the location of the lung. While techniques such as data augmentation can minimize the impact of translation, scaling, and rotation on a neural network's performance, explicitly removing these sources of variation can improve performance [45]. We will evaluate two approaches for their ability to identify the lungs location within an X-ray image. The first model's architecture is a variant of the VGG (Visual Geometry Group) architecture, but with residual network blocks for pixel-level object detection. This has previously been shown to work well for adult lung segmentation [44,46], in combination with image histogram equalization. This architecture of this lung detection model will be determined from a hyper-parameter search, which will match the model's complexity to the lung detection problem. The lung detection model will initially be trained on 300 paediatric chest X-ray images and evaluated on a validation set of an additional 100 paediatric chest X-ray images and assessed using the Dice coefficient to evaluate the model's performance. Additional images may be used if the model's performance is deemed insufficient. In line with observations by Dai and colleagues [44], 300 images should be sufficient for our lung segmentation and centering task. Key point detection on the segmented images by Binary Robust Invariant Scalable Keypoints (BRISK) [47] or Fast Retina Keypoint (FREAK) [48] to align the images to a reference image using the Open Source Computer Vision Library (OpenCV), an image manipulation toolkit [49]. The second approach for lung detection will be to explore the use of spatial transformer networks [45] in conjunction with object recognition deep learning architectures, thereby enabling a single model to detect the lung and diagnose paediatric pneumonia.

For peer review only

The second stage of the software tool will diagnose pneumonia from the aligned paediatric chest X-ray images. This identification task will be performed in a multi-task prediction setting, namely that the CNN's goal will be to predict 1) primary end-point pneumonia, 2) other lung infiltrates, and/or 3) pleural fluid in both the left and right lung for a total of 6 binary labels. The pneumonia diagnosis model will be based on the DenseNet architecture [25]. Dilated convolutions [50] will be incorporated into the architecture to aggregate features spanning different distances across the lungs. The final model architecture will be determined from an architecture hyper parameter search over the number of convolution layers per dense block, feature-map growth rate, and feature-map compression will be explored. The number of dense blocks will be kept fixed to four. The binary cross entropy loss function will be used for model training, to assess differences between the model's prediction and the ground truth provided by the labels and to guide how the model parameters are to be updated to improve the model predictions. An ensemble of five models will form the final predictor, whose predictions for each of the 6 output classes will be the unweighted average of the five models' predicted class probabilities.

Statistical Methods and Performance Evaluation

To evaluate the quality of the labels associated with the ~10,000 chest X-ray images, we will evaluate the readers' concordance with each other and with the compiled multi-reader labels using Cohen's kappa coefficient, sensitivity, and specificity for both the right and left lung. 95% confidence intervals will be assessed empirically by bootstrapping.

To select the model hyper-parameters (model complexity), the data will be partitioned into 5-folds and tested on 10% of the data. This will allow us to train models on 72% (4 folds) and validate on 18% (1 fold) of the data and to compare models with different hyper-parameters. Given the prevalence of pneumonia within this dataset measured to date (29%), having enough positive samples to validate the model for both the right and left lung is a concern, which we believe a 5-fold cross validation addresses. Hyper-parameters will be determined with a mixture of grid searching and prior literature values. These five models will form an ensemble

For peer review only

and the final predictions for each of the 6 output classes will be formed by taking the unweighted average of the predicted class probabilities for each label.

Two external image datasets (objective 3) and 10% of the data derived from primary 10,000 image dataset will be used to evaluate the software tool. The first external test set will be from KWMCH, whose labels will be provided by the sa me readers who labelled the training image dataset. This dataset will enable the study to evaluate the impact of using different X-ray imagining machines and technicians and disease prevalence. The second external test dataset will be from WHO CRES, whose images were independently collected and independently labelled by clinicians and radiologists following the WHO Radiological Pneumonia definitions [4]. This will assess how transferable our method will be to other study sites. Performance will be evaluated using the area under the receiver operator curve (auROC), the area under the precision-recall curve (auPR), sensitivity, and specificity. A confusion matrix will also be provided. Finally, 95% confidence intervals will be assessed empirically by bootstrapping. Benchmark dataset will be resampled with replacement 1000 times.

For peer review only

Patient and Public Involvement

No patient or members of the public were involved in this study.

DISCUSSION

The study will be the first to use a large dataset whose labels are derived from multiple expert readers to train a deep-learning model to diagnose paediatric primary-end point pneumonia. Prior work to identify pneumonia in adult lungs using a CNN achieved a performance of 0.931 auROC and a 0.815 auROC on an independently collected benchmark. This computational model was trained on chest X-ray images labelled from a single reader whose pneumonia labels were extracted from radiology reports via a NLP system that may introduce extraction errors [12]. A prior study that leverage multiple expert readers, was small in size, containing 858

For peer review only - http://bmjopen.bmj.com/site/about/guidelines.xhtml

pediatric chest X-ray images interpreted for PEP by three readers [2]. Since the X-rays were collected over a short period of time, this dataset likely lacks the image variance observed in LMIC settings. The study authors used a SVM to make pixel level predictions and a 95th percentile pixel score as an image score to achieve a 0.850 auROC [13]. We are expecting our model to have similar or better performance since the training data is larger in size and evaluated by multiple readers. Furthermore, the radiological pneumonia definition is broad in comparison to the more precisely defined WHO of diagnosing primary-end point pneumonia.

A limitation of the study is the use of chest X-ray images from individuals ≤ 60 months of age derived from a single institution using two machines to capture the images to construct a computational model to interpret chest X-ray images for the presence of pneumonia. Findings from the study may not generalize to individuals ≥60 months of age. However, this is the most appropriate age group for this study as more than 90% of pediatric pneumonia cases are limited to children <5 years of age.

While the computational model will be evaluated against an independently collected and labelled chest X-ray image dataset for the presence or absence of primary end-point pneumonia (WHO CRES dataset), some findings may not generalize to other settings (as the training data is from a single institution). To overcome this limitation, we are aiming to extend this study as a multi-country surveillance, if successful.

For peer review only

Data Availability

All findings will be published in an open-access journal. All X-ray labels and statistical code will be made openly available. The model and images will be made available upon request.

Acknowledgements

This research was commissioned by the UK National Institute for Health Research (NIHR) Global Health Research Unit on Respiratory Health (RESPIRE), using UK Aid from the UK Government. The views expressed in this publication are those of the author(s) and not necessarily those of the NIHR or the UK Department of Health and Social Care. We are grateful to Mohammad Shahidul Islam and Md. Shariful Islam for the intellectual and technical guidance.

Author's Contributors

Conceptual construction of research and design: MGFS, SS, and SKS. Critical manuscript review: MGFS, SS, SKS, SAS, SL and HN. All authors have approved the final version of the manuscript for submission for publication.

Funding

This work has received funding support from NIHR Global Health Research Unit on Respiratory Health (RESPIRE) 16/136/109.

Competing interests

None declared

For peer review only

Ethics

All study protocols were approved by the Ethical Review Committees of the Bangladesh Institute of Child Health.

References

1 McAllister DA, Liu L, Shi T, *et al.* Global, regional, and national estimates of pneumonia morbidity and mortality in children younger than 5 years between 2000 and 2015: a systematic analysis. *Lancet Glob Health* 2019;**7**:e47–57.

2 O'Brien KL, Baggett HC, Brooks WA, *et al.* Causes of severe pneumonia requiring hospital admission in children without HIV infection from Africa and Asia: the PERCH multi-country case-control study. *The Lancet* 2019.

3 National Institute of Population Research and Training. BANGLADESH DEMOGRAPHIC AND HEALTH SURVEY. 2014.https://dhsprogram.com/pubs/pdf/FR311/FR311.pdf

4 Cherian T, Mulholland EK, Carlin JB, *et al.* Standardized interpretation of paediatric chest radiographs for the diagnosis of pneumonia in epidemiological studies. *Bull World Health Organ* 2005;**83**:353–9.

5 Elemraid MA, Muller M, Spencer DA, *et al.* Accuracy of the interpretation of chest radiographs for the diagnosis of paediatric pneumonia. *PLoS One* 2014;**9**:e106051.

6 McCollum ED, Ahmed S, Chowdhury NH, *et al.* Chest radiograph reading panel performance in a Bangladesh pneumococcal vaccine effectiveness study. *BMJ Open Respir Res* 2019;**6**:e000393.

7 Liu Y, Gadepalli K, Norouzi M, *et al.* Detecting cancer metastases on gigapixel pathology images. *ArXiv Prepr ArXiv170302442* 2017.

8 McKinney SM, Sieniek M, Godbole V, *et al.* International evaluation of an AI system for breast cancer screening. *Nature* 2020;**577**:89–94.

9 Lakhani P, Sundaram B. Deep learning at chest radiography: automated classification of pulmonary tuberculosis by using convolutional neural networks. *Radiology* 2017;**284**:574–82.

10 Gulshan V, Peng L, Coram M, *et al.* Development and validation of a deep learning algorithm for detection of diabetic retinopathy in retinal fundus photographs. *Jama* 2016;**316**:2402–10.

11 Rajpurkar P, Irvin J, Zhu K, *et al.* Chexnet: Radiologist-level pneumonia detection on chest x-rays with deep learning. *ArXiv Prepr ArXiv171105225* 2017.

For peer review only

12 Zech JR, Badgeley MA, Liu M, *et al.* Variable generalization performance of a deep learning model to detect pneumonia in chest radiographs: A cross-sectional study. *PLoS Med* 2018;**15**:e1002683.

13 Mahomed N, van Ginneken B, Philipsen RHHM, *et al.* Computer-aided diagnosis for World Health Organization-defined chest radiograph primary-endpoint pneumonia in children. *Pediatr Radiol* 2020;**50**:482–91. doi:10.1007/s00247-019-04593-0

14 Rajpurkar P, Irvin J, Ball RL, *et al.* Deep learning for chest radiograph diagnosis: A retrospective comparison of the CheXNeXt algorithm to practicing radiologists. *PLoS Med* 2018;**15**:e1002686.

15 Russakovsky O, Deng J, Su H, *et al.* Imagenet large scale visual recognition challenge. *Int J Comput Vis* 2015;**115**:211–52.

16 Shin H-C, Roth HR, Gao M, *et al.* Deep convolutional neural networks for computer-aided detection: CNN architectures, dataset characteristics and transfer learning. *IEEE Trans Med Imaging* 2016;**35**:1285–98.

17 Rajaraman S, Candemir S, Kim I, *et al.* Visualization and interpretation of convolutional neural network predictions in detecting pneumonia in pediatric chest radiographs. *Appl Sci* 2018;**8**:1715.

18 Stephen O, Sain M, Maduh UJ, *et al.* An efficient deep learning approach to pneumonia classification in healthcare. *J Healthc Eng* 2019;**2019**.

19 Kermany DS, Goldbaum M, Cai W, *et al.* Identifying medical diagnoses and treatable diseases by image-based deep learning. *Cell* 2018;**172**:1122–31.

20 Rahman T, Chowdhury ME, Khandakar A, *et al.* Transfer learning with deep convolutional neural network (CNN) for pneumonia detection using chest X-ray. *Appl Sci* 2020;**10**:3233.

21 Ucar F, Korkmaz D. COVIDiagnosis-Net: Deep Bayes-SqueezeNet based diagnosis of the coronavirus disease 2019 (COVID-19) from X-ray images. *Med Hypotheses* 2020;**140**:109761.

22 Chouhan V, Singh SK, Khamparia A, *et al.* A novel transfer learning based approach for pneumonia detection in chest X-ray images. *Appl Sci* 2020;**10**:559.

23 Hashmi MF, Katiyar S, Keskar AG, *et al.* Efficient pneumonia detection in chest xray images using deep transfer learning. *Diagnostics* 2020;**10**:417.

24 Krizhevsky A, Sutskever I, Hinton GE. Imagenet classification with deep convolutional neural networks. *Commun ACM* 2017;**60**:84–90.

For peer review only

25 Huang G, Liu Z, Van Der Maaten L, *et al.* Densely connected convolutional networks. In: *Proceedings of the IEEE conference on computer vision and pattern recognition*. 2017. 4700–8.

26 Szegedy C, Vanhoucke V, Ioffe S, *et al.* Rethinking the inception architecture for computer vision. In: *Proceedings of the IEEE conference on computer vision and pattern recognition*. 2016. 2818–26.

27 Szegedy C, Liu W, Jia Y, *et al.* Going deeper with convolutions. In: *Proceedings of the IEEE conference on computer vision and pattern recognition*. 2015. 1–9.

28 He K, Zhang X, Ren S, *et al.* Deep residual learning for image recognition. In: *Proceedings of the IEEE conference on computer vision and pattern recognition*. 2016. 770–8.

29 Vaswani A, Shazeer N, Parmar N, *et al.* Attention is all you need. *ArXiv Prepr ArXiv170603762* 2017.

30 Cha S-M, Lee S-S, Ko B. Attention-Based Transfer Learning for Efficient Pneumonia Detection in Chest X-ray Images. *Appl Sci* 2021;**11**:1242.

31 Toğaçar M, Ergen B, Cömert Z, *et al.* A deep feature learning model for pneumonia detection applying a combination of mRMR feature selection and machine learning models. *Irbm* 2020;**41**:212–22.

32 Zhao W, Wang L, Zhang Z. Artificial ecosystem-based optimization: a novel nature-inspired meta-heuristic algorithm. *Neural Comput Appl* 2019;:1–43.

33 Sahlol AT, Abd Elaziz M, Tariq Jamal A, *et al.* A novel method for detection of tuberculosis in chest radiographs using artificial ecosystem-based optimisation of deep neural network features. *Symmetry* 2020;**12**:1146.

34 Sahlol AT, Yousri D, Ewees AA, *et al.* COVID-19 image classification using deep features and fractional-order marine predators algorithm. *Sci Rep* 2020;**10**:1–15.

35 Zhang J, Xie Y, Pang G, *et al.* Viral Pneumonia Screening on Chest X-rays Using Confidence-Aware Anomaly Detection. *IEEE Trans Med Imaging* 2020.

36 Ke Q, Zhang J, Wei W, *et al.* A neuro-heuristic approach for recognition of lung diseases from X-ray images.

37 Mirjalili S. Moth-flame optimization algorithm: A novel nature-inspired heuristic paradigm. *Knowl-Based Syst* 2015;**89**:228–49.

38 Mirjalili S. The ant lion optimizer. *Adv Eng Softw* 2015;**83**:80–98.

For peer review only

39 WHO Chest Radiography in Epidemiological Studies (CRES). https://who-cres.mcri.edu.au/

40 Mahomed N, Fancourt N, De Campo J, *et al.* Preliminary report from the World Health Organisation chest radiography in epidemiological studies project. *Pediatr Radiol* 2017;**47**:1399–404.

41 Hasan AZ, Saha S, Saha SK, *et al.* Using pneumococcal and rotavirus surveillance in vaccine decision-making: A series of case studies in Bangladesh, Armenia and the Gambia. *Vaccine* 2018;**36**:4939–43. doi:10.1016/j.vaccine.2018.06.001

42 Global Invasive Bacterial Vaccine-Preventable Diseases Surveillance — 2008–2014. https://www.cdc.gov/mmwr/preview/mmwrhtml/mm6349a4.htm (accessed 2 Sep 2020).

43 Saha SK, Hossain B, Islam M, *et al.* Epidemiology of invasive pneumococcal disease in Bangladeshi children before introduction of pneumococcal conjugate vaccine. *Pediatr Infect Dis J* 2016;**35**:655–61.

44 Dai W, Dong N, Wang Z, *et al.* Scan: Structure correcting adversarial network for organ segmentation in chest x-rays. In: *Deep Learning in Medical Image Analysis and Multimodal Learning for Clinical Decision Support*. Springer 2018. 263–73.

45 Jaderberg M, Simonyan K, Zisserman A, *et al.* Spatial transformer networks. In: *Advances in neural information processing systems*. 2015. 2017–25.

46 Bansal A, Chen X, Russell B, *et al.* Pixelnet: Towards a general pixel-level architecture. *ArXiv Prepr ArXiv160906694* 2016.

47 Leutenegger S, Chli M, Siegwart RY. BRISK: Binary robust invariant scalable keypoints. In: *2011 International conference on computer vision*. Ieee 2011. 2548–55.

48 Alahi A, Ortiz R, Vandergheynst P. Freak: Fast retina keypoint. In: *2012 IEEE Conference on Computer Vision and Pattern Recognition*. Ieee 2012. 510–7.

49 Bradski G. The OpenCV Library. *Dr Dobbs J Softw Tools* 2000.

50 Yu F, Koltun V. Multi-scale context aggregation by dilated convolutions. *ArXiv Prepr ArXiv151107122* 2015.

FIGURE LEGENDS

Figure 1: Study design schema. PEP: Primary end point pneumonia; KWMCH : Kumudini Women's Medical College Hospital

For peer review only

Figure 2: Digitized chest X-ray images include (Left) full body images and (Right) images of just the lung area.

For peer review only

For peer review only - http://bmjopen.bmj.com/site/about/guidelines.xhtml

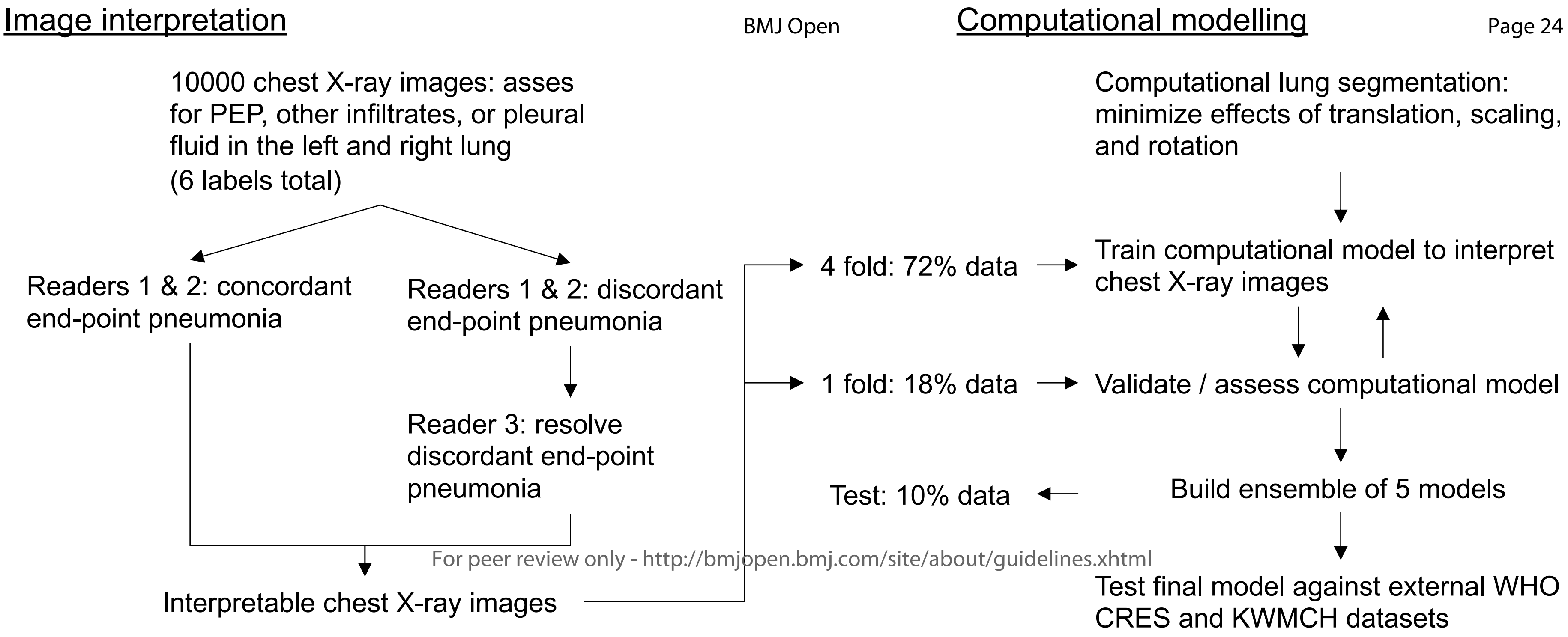

For peer review only - http://bmjopen.bmj.com/site/about/guidelines.xhtml

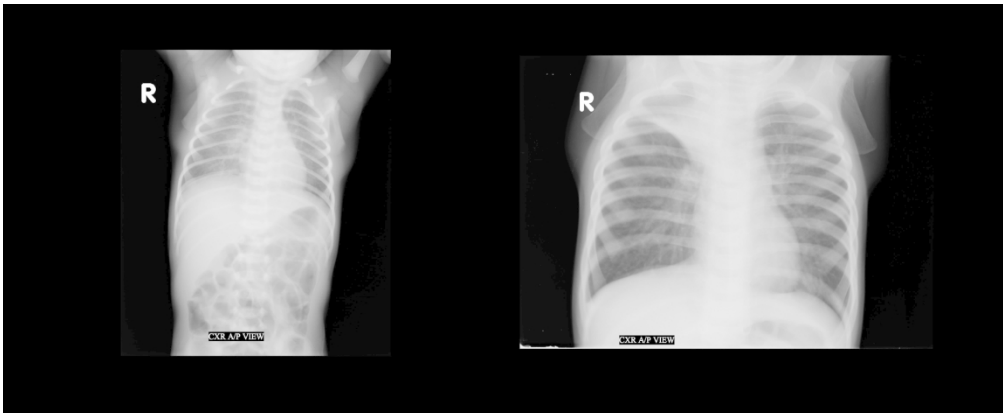

Figure 2: Digitized chest X-ray images include (Left) full body images and (Right) images of just the lung area.

BMJ Open

A study protocol and design for the assessment of paediatric pneumonia from X-Ray images using deep learning

Journal:	*BMJ Open*
Manuscript ID	bmjopen-2020-044461.R3
Article Type:	Protocol
Date Submitted by the Author:	28-Feb-2021
Complete List of Authors:	Sun, Mark; Child Health Research Foundation<br>Saha, Senjuti; Child Health Research Foundation; Johns Hopkins University Bloomberg School of Public Health,<br>Shah, Syed Ahmar; The University of Edinburgh Usher Institute of Population Health Sciences and Informatics,<br>Luz, Saturnino; University of Edinburgh School of Molecular Genetic and Population Health Sciences, Usher Institute of Population Health Sciences and Informatics<br>Nair, Harish; University of Edinburgh School of Molecular Genetic and Population Health Sciences,<br>Saha, Samir; Child Health Research Foundation
**Primary Subject Heading**:	Diagnostics
Secondary Subject Heading:	Diagnostics, Health informatics, Infectious diseases, Public health, Respiratory medicine
Keywords:	Paediatric radiology < PAEDIATRICS, Respiratory infections < THORACIC MEDICINE, Public health < INFECTIOUS DISEASES, Chest imaging < RADIOLOGY & IMAGING, Diagnostic radiology < RADIOLOGY & IMAGING

SCHOLARONE™
Manuscripts

I, the Submitting Author has the right to grant and does grant on behalf of all authors of the Work (as defined in the below author licence), an exclusive licence and/or a non-exclusive licence for contributions from authors who are: i) UK Crown employees; ii) where BMJ has agreed a CC-BY licence shall apply, and/or iii) in accordance with the terms applicable for US Federal Government officers or employees acting as part of their official duties; on a worldwide, perpetual, irrevocable, royalty-free basis to BMJ Publishing Group Ltd ("BMJ") its licensees and where the relevant Journal is co-owned by BMJ to the co-owners of the Journal, to publish the Work in this journal and any other BMJ products and to exploit all rights, as set out in our licence.

The Submitting Author accepts and understands that any supply made under these terms is made by BMJ to the Submitting Author unless you are acting as an employee on behalf of your employer or a postgraduate student of an affiliated institution which is paying any applicable article publishing charge ("APC") for Open Access articles. Where the Submitting Author wishes to make the Work available on an Open Access basis (and intends to pay the relevant APC), the terms of reuse of such Open Access shall be governed by a Creative Commons licence – details of these licences and which Creative Commons licence will apply to this Work are set out in our licence referred to above.

Other than as permitted in any relevant BMJ Author's Self Archiving Policies, I confirm this Work has not been accepted for publication elsewhere, is not being considered for publication elsewhere and does not duplicate material already published. I confirm all authors consent to publication of this Work and authorise the granting of this licence.

For peer review only

A study protocol and design for the assessment of paediatric pneumonia from X-Ray images using deep learning

Mark George Ford Sun*[1], Senjuti Saha*[1,2,#], Syed Ahmar Shah[5], Saturnino Luz[3,5], Harish Nair[4,5], Samir K Saha[1,4,#]

[1]Child Health Research Foundation, Dhaka, Bangladesh

[2]Johns Hopkins Bloomberg School of Public Health, USA

[3]Centre for Global Health, Usher Institute, University of Edinburgh

[4]Dhaka Shishu (Children) Hospital, Bangladesh

[5]NIHR Global Health Research Unit on Respiratory Health (RESPIRE), Usher Institute, University of Edinburgh, Edinburgh, UK

*These authors made equal contribution to the paper

#Co-correspondence to:

Prof Samir K Saha, PhD, FRCPath
Dhaka Shishu Hospital & Bangladesh Institute of Child Health
Child Health Research Foundation
23/2 Sel Huq Skypark, Block-B Khilji Road, Dhaka 1207, Bangladesh

Senjuti Saha, PhD
Child Health Research Foundation
23/2 Sel Huq Skypark, Block-B Khilji Road, Dhaka 1207, Bangladesh
senjutisaha@chrfbd.org; +88 01711646038

samir@chrfbd.org; +8801713461254

Word count: 300 (abstract); 3,248 (main text)

For peer review only

ABSTRACT

Introduction

In low and middle-income countries, pneumonia remains the leading cause of illness and death in children <5 years. The recommended tool for diagnosing paediatric pneumonia is the interpretation of chest X-ray images, which is difficult to standardize and requires trained clinicians/radiologists. Current automated computational tools have primarily focused on assessing adult pneumonia and were trained on images evaluated by a single specialist. We aim to provide a computational tool using a deep-learning approach to diagnose paediatric pneumonia using X-ray images assessed by multiple specialists trained by the WHO expert X-ray image reading panel.

Methods and analysis

Approximately 10,000 paediatric chest X-ray images are currently being collected from an ongoing WHO-supported surveillance study in Bangladesh. Each image will be read by two trained clinicians/radiologists for the presence or absence of primary end-point pneumonia (PEP) in each lung, as defined by the WHO. Images whose PEP labels are discordant in either lung will be reviewed by a third specialist and the final assignment will be made using a majority vote. Convolutional neural networks will be used for lung segmentation to align and scale the images to a reference, and for interpretation of the images for the presence of PEP. The model will be evaluated against an independently collected and labelled set of images from the WHO. The study outcome will be an automated method for the interpretation of chest radiographs for diagnosing pediatric pneumonia.

Ethics and Dissemination

All study protocols were approved by the Ethical Review Committees of the Bangladesh Institute of Child Health, Bangladesh. The study sponsor deemed it unnecessary to attain ethical approval from the Academic and Clinical Central Office for Research and Development of University of Edinburgh, UK. The study uses existing X-ray images from an ongoing WHO-coordinated surveillance. All findings will be published in an open-access journal. All X-ray labels

For peer review only

and statistical code will be made openly available. The model and images will be made available upon request.

Strengths and limitations of this study

- The main strength of the study is that it will employ a large, high confidence dataset of ~10,000 paediatric chest X-ray images interpreted by multiple trained readers for the presence of primary endpoint pneumonia.
- Another strength is that the computational tool will be evaluated against two external paediatric chest X-ray image datasets to assess generalizability of the model.
- The primary limitation of the study is that the computational model will be trained from chest X-ray images derived from a single study site.

For peer review only

INTRODUCTION

Paediatric pneumonia overview

Pneumonia is a leading cause of childhood illness and death in developing countries. Globally, about 138 million cases and 0.9 million deaths are estimated to occur annually due to pneumonia with about six million cases in Bangladesh alone [1]. However, determining the disease prevalence is complicated due to the lack of reliable diagnostic tools: 1) blood cultures are insensitive as most cases are likely not bacteremic and therefore a blood culture cannot determine a bacterial respiratory infection and 2) other diagnostic procedures like radiological findings are difficult to standardize. While the true proportions of pneumonia etiologies (viral/bacterial) are unknown [2], 34% of children in Bangladesh diagnosed with pneumonia receive empirical antibiotics [3]. Image based diagnostics such as the interpretation of chest X-ray images to identify paediatric pneumonia are regarded as the “gold standard” [4], but they are difficult to standardize and the interpretations can vary between radiologists and clinicians [5,6]. Furthermore, the limited number of well-trained radiologists and clinicians limits the use of X-ray images as a ubiquitous diagnostic tool for pneumonia. This lack of accurate diagnosis hampers the determination of the true burden of pneumonia, and the proportion that is preventable by vaccination, which in turn impedes institution of evidence-based intervention strategies and vaccine impact measurements.

Deep learning methods such as convolutional neural networks (CNNs) may be used to expedite the adoption of chest X-ray images for the diagnosis of pneumonia. CNNs are composed of multiple feature extractors stacked on top of each other. During the training process the feature extractors near the model input learn primitive image features such as edges, whereas feature extractors deeper into the model learn domain specific relationships important for the diagnostic task. CNNs have successfully been applied to assist in the detection and classification of human diseases like breast cancer [7,8], tuberculosis [9], diabetic retinopathy [10] and pneumonia [11–13] directly from images. In some instances, these systems performed as well as trained health professionals. While systems have been developed to identify paediatric

For peer review only

pneumonia from X-ray images, the datasets used for training are either small (<1000), evaluated on X-ray images derived from the same study site, or don't compare the model's performance with respect to a clinician/radiologist. Our goal is to construct a computational framework to automatically and systematically interpret paediatric chest X-ray images to diagnose pneumonia using ~10 000 training images and whose performance both generalized across datasets and is benchmarked to that of a clinician / radiologist. If successful, an automated system to detect pediatric pneumonia will have significant public health benefits. It will accelerate the adoption of chest X-ray images in low- and middle-income country (LMIC) settings, where there is a lack of trained radiologists and clinicians, and contribute to the standardization of X-ray image analysis. This will encourage clinicians to rationalise the use of antibiotic therapies and to facilitate antibiotic stewardship.

Diagnosing paediatric pneumonia

Chest X-ray images interpreted to have primary endpoint pneumonia (PEP) are considered the "gold standard" for the diagnosis of paediatric pneumonia [4]. The PEP case definition was developed by the WHO as part of clinical trials to assess the efficacy of vaccines against *Streptococcus pneumoniae* and *Haemophilus influenza* type b (Hib) and is defined as the presence of end-point consolidation or pleural effusion [4]. Despite efforts to standardize the interpretation of chest radiographs by training specialists to the WHO definitions, significant discrepancies are still observed between readers interpreting the same chest X-ray images, due to the subjective nature of the diagnosis [5,6]. To overcome differences between trained readers, a panel of multiple readers may be employed to interpret images [6].

Establishing well-funded and rigorous clinical trials with multiple trained readers to evaluate every chest X-ray image is an onerous task. This time intensive task may be alleviated with the aid of computational tools to diagnose pneumonia directly from chest X-ray images. CNN models are one such tool, but require thousands of labelled images to successfully be trained from a randomly initialized state. To this end, adult radiology reports have been analyzed by

For peer review only

natural language processing (NLP) systems to programmatically assign labels. This enabled the collection of a large computationally labelled chest X-ray images (40,000 – 110,000 images) for the training of CNN models to diagnose pneumonia directly from adult chest X-ray images [11,12,14]. Transfer learning can be applied to reduce model training time or to enable model training should the dataset be too small to train a model from a randomly initialized state. Models are first pretrained on a large image dataset such as ImageNet [15], containing more than a million labelled images of common entities. These pretrained models are transferred to the domain of interest by finetuning the model [16]. Zech and colleagues found that transfer learning for a large adult chest X-ray image dataset achieved a performance of 0.931 for the area under the receiver operator curve (auROC). When evaluated on an external benchmark dataset, whose data source differs from that of the training dataset, the model performed significantly worse with an auROC of 0.815. This performance degradation of models trained on a single site was found in 3 out of 5 instances [12], highlighting the need of external evaluation datasets to avoid the reporting of inflated performance metrics, which likely holds true in the paediatric pneumonia setting.

Model training from randomly initialize weights [17,18] and model finetuning has been applied for the diagnosis of paediatric pneumonia directly from X-ray images [19–21]. Efforts to increase performance within the paediatric pneumonia setting have involved using multiple different architectures to form model ensembles, thereby expanding the space of functions that can be learned to associate an X-ray image to a diagnosis label [22,23]. Chouhan and colleagues used transfer learning with five different models [22] based on the AlexNet [24], DenseNet-121 [25], Inception V3 [26], GoogLeNet [27], and ResNet18 [28] architectures, for a pediatric pneumonia X-ray image dataset containing 5856 labelled images [19]. Combining the individual model predictions by a majority vote (99.34 auROC) [22] or in a linear combination scheme (99.76 auROC) [23] were suggestive that a performance increase relative to a single model is possible. Rather than combining the final scalar prediction scores, multiple CNN models may be treated as feature extractors that may be used as input to an attention mechanism, enabling the model to attend to import features. Cha and colleagues used the Self-

For peer review only

Attention mechanism [29] over the ensemble feature vectors to achieve an auROC of 96.03 [30]. Feature selection may be applied over the ensemble feature vector using the minimum redundancy maximum relevance (mRMR) method. The selected features may be used as input into a classifier such as a support vector machine (SVM) [31]. Other feature selection schemes include the use of Artificial Ecosystem-based Optimization (AEO) [32,33] and the Marine Predators Algorithm (MPA) enhanced by fractional-order calculus (FO) [34] , which have respectively been applied for the diagnosis of tuberculosis and COVID-19 from X-ray images. Alternatively, the prediction of a CNN may be gated by an anomaly detection scheme using a modified loss function to guide model training [35]. These methods were evaluated on images from the same study site, thus additional investigation is required to ensure that the models are learning discriminatory radiological features of pneumonia and are not exploiting confounding features of the study site.

During CNN model training, image features responsible for the radiological diagnosis of pneumonia are learned. Rather than learning the image features directly from the training dataset, images features may be explicitly specified. Ke and colleagues used global image descriptors of hue, saturation, and brightness as input to a fully connected neural network to predict pneumonia [36]. Representative pixels of disease lung tissue were identified using the Moth-Flame [37] and Ant Lion [38] heuristics. Global image descriptors are too coarse of a feature to predict pixel level properties. Mahomed and colleagues used Gaussian derivative filters to specify texture features on a per pixel level and used a support vector machine (SVM) for classification. A small pediatric dataset of 858 chest X-ray images was interpreted for PEP by three readers [2] was used to train there model, which achieved a 0.850 auROC [13]. Our study will use 10,000 images interpreted for PEP. Furthermore, by specifying the features that are to be used by a computational prediction method, one may potentially omit features that can be directly learned from chest X-ray images, which will likely negatively impact model performance.

For peer review only

In light of the grading discrepancies found between readers interpreting pediatric chest X-ray images for pneumonia, our study will use multiple readers trained by the expert WHO X-ray image reading panel. Additionally, all readers will be blinded to the labels provided by the other readers. Furthermore, our study will use X-ray images collected from an LMIC hospital by radiologists for patient services. These images, collected over a period of 8 years, were not part of any specific radiological study. Hence, technologists taking the images were not uniformly trained specifically for this study, reflecting the variability commonly found in most hospitals. To demonstrate that our proposed system generalizes to other study sites, we will benchmark the system against images collected from two different sources: 1) the Kumudini Women's Medical College and Hospital (KWMCH) and 2) the WHO Chest Radiography in Epidemiological Studies (WHO CRES) working group [39].

Objectives

We aim to build a computational tool using a deep-learning approach to diagnose paediatric pneumonia using X-ray images assessed by multiple WHO-trained specialists. The primary project objectives are to:

(1) Collect and label ~10,000 paediatric chest X-rays images with high confidence, indicating if the right and left lung are healthy or if either lung meets the primary-end point pneumonia criteria
(2) Develop a software tool leveraging advances in the field of image recognition, in particular deep learning architectures and strategies for training these models, to diagnose paediatric pneumonia from chest X-ray images
(3) Evaluate the model against ~100 images collected from an alternative site labelled by the same readers of this study and against 130 independently collected images from sites differing from those described in this study a [39,40].

For peer review only

METHODS AND ANALYSIS

The overall design of the study is illustrated in Figure 1.

Participants and data source

Chest X-ray images will be derived from the ongoing WHO-coordinated Invasive Bacterial Vaccine Preventable Diseases (IB-VPD) Surveillance Platform in Bangladesh [41,42]. This surveillance system monitors burden of bacterial pneumonia, meningitis, and sepsis in children under 5 years old in-patients, using protocols described elsewhere [43]. Chest X-ray images are performed on the advice of the treating physicians Digital Imaging and Communications in Medicine (DICOM) images are collected by our team periodically. Technicians performing the chest X-rays are not trained for this specific project avoiding any bias in picture quality. Collection of the chest X-ray images started in 2013 and we expect to reach 10,000 chest X-ray images in 2021.

Two machines are used to capture the images: 1) SG Healthcare Jumong General and 2) TRIUP International Corp TRF100. The intent is for 100 images derived from the Kumudini Women's Medical College and Hospital (KWMCH), also an IB-VPD Surveillance Platform partner, to be included as a test set to assess the impact of images collected from different X-ray machines and technicians. The KWMCH site has two machines to capture images: 1) Hitachi Radnext 50 and 2) General Electric Dx-525. In the IB-VPD surveillance , written consent is obtained from parents or caregivers of all participants for other aspects of the study, including collection of data and additional analysis. Images of any participants that withdraw the consent from the surveillance study will not be considered for this study. The data generated from the individual patients will be deidentified prior to analysis.

For peer review only

Inclusion Criteria

An X-ray image will be included in this study if

- The age of the child for whom the X-ray is performed is <60 months,
- The X-ray was performed in one of the two study hospitals and the child was enrolled in the IB-VPD surveillance study, and
- The X-ray image captures the lung area

The inclusion criteria for the IB-VPD surveillance system for pneumonia are:

- The age of the child is <60 months,
- The child had coughing or difficulty breathing and tachypnea when calm at a rate of ≥60 breaths/min in an infant aged <2 months, or
- ≥50 breaths/min in an infant aged 2 to <12 months, or ≥40 breaths/min in children ≥12 months

Exclusion Criteria

Any image that is marked “uninterpretable” (features of the images are not interpretable with respect to presence or absence of primary end-point pneumonia) by the first two readers.

Sample size

In this study, we are attempting to train computational models rather than to assess if two populations differ. For this reason, we turn to literature to estimate the training dataset sizes required to build the proposed software tool to interpret paediatric chest X-rays. The tool to diagnose paediatric pneumonia (objective 2) will be composed of two stages: 1) lung detection to align the chest X-ray images to a reference image and 2) interpretation of paediatric chest X-rays for the presence of pneumonia.

For peer review only

To identify the location of lungs within the X-ray image, Dai and colleagues developed a generative adversarial network (GAN) and a baseline residual block network. They report Dice coefficient values of 97.3% and 96.3% respectively, where the Dice coefficient is a measure of similarity between the predicted location and a reference mask, with just 247 adult chest X-rays images [44]. The results will likely translate to the proposed study as we are interested in the identification of the same gross anatomical structures. This suggests that detecting the lung's location within paediatric chest X-rays can readily be achieved with training using only a small proportion (about 300 from 10,000) of the collected chest X-ray images.

For interpretation of chest X rays, we will focus on studies that used expert panels to label X-ray images as a means to estimate the dataset size required to train a CNN to diagnose paediatric pneumonia directly from chest X-ray images. Prior studies used NLP systems to analyze radiology reports to programmatically assign labels, enabling large collections of chest X-ray images to be labelled [11,12]. However, due to the variability found between trained readers when diagnosing paediatric pneumonia from chest X-ray images [5,6], we believe that a more appropriate strategy is to have each image assessed by multiple readers to ensure a high quality training dataset. Recent deep learning methods have diagnosed diseases such as tuberculosis after training on 1,007 chest X-ray images with a performance of 0.99 for the area under the receiver operator curve (auROC) [9] and diabetic retinopathy with a performance of 0.974 auROC using 128,175 retinal training images (28.1% were classified as referable diabetic retinopathy) [10]. For the diagnosis of referable diabetic retinopathy, Gulshan and colleagues found that using 10,000 retinal images to train their model resulted in a sensitivity and specificity respectively of 97% and 75% with saturation occurring at 60,000 images with a sensitivity and specificity respectively of 97.5% and 93.4%. We noted that the performance found between a reading panel and images labelled by an expert reader with a sensitivity and specificity respectively of 77% and 96.3% for paediatric pneumonia [6].

For peer review only

To date 8,002 paediatric chest X-ray images have been reviewed by a single clinician, of which 2,320 (29%) were identified with primary end-point pneumonia in either lung, 4786 (60%) had no pneumonia, and 898 (11%) were uninterpretable. We expect this class ratio to remain true for the complete dataset. Together, these studies and observations suggest that the 10,000 paediatric chest X-rays being collected will result in a suitable dataset to train a neural network to identify pneumonia cases among paediatric chest X-ray images.

Study Procedure

To build a software tool capable of diagnosing paediatric pneumonia directly from X-ray images, it is first necessary to establish a representative training dataset (Objective 1) that is of a sufficiently large size for CNN to be successfully trained (Figure 1). To this end, ~10,000 paediatric chest X-rays will be collected and labelled by radiologists/clinicians standardized to the WHO definitions (each blinded to all labels provided by the other readers) [4] who will interpret the images as having 1) primary end-point pneumonia (PEP), 2) other lung infiltrates, and/or 3) pleural fluid in either the left or right lung, for a total of six possible binary outcomes. Multiple labels for both lungs rather than a single binary value will enable information from both lungs to be leveraged and information about other non-PEP classes may be used to guide the pneumonia prediction task. A challenge to assigning a pneumonia label to an image is the high discordance observed between diagnoses from trained radiologists / clinicians [5]. We plan on mitigating this by having multiple readers standardized to the WHO definitions to read each chest X-ray image. Specifically, two primary readers will assess all images meeting the described inclusion criteria. Chest X-ray images having discordant PEP labels will be reassessed by a third reader. A simple majority voting will be used to assign the multiclass labels for images. The remaining discordant labels (ie. not PEP) will be assigned randomly to reflect uncertainty in the label. Since “uninterpretable” images may still have evidence of some of the six outcomes, images labelled as “uninterpretable” by both readers will be excluded from the computational modeling process. Images labelled as “uninterpretable” by one primary reader but “interpretable” by the other will be re-evaluated by the third reader (Figure 1).

For peer review only

The software tool to diagnose paediatric pneumonia (objective 2) will be composed of two stages: 1) lung detection to align the chest X-ray images to a reference image and 2) interpretation of paediatric chest X-ray images for the presence of pneumonia. Chest X-ray images of paediatric patients can be highly variable, specifically in resource poor settings without well-trained personnel and/or high patient flow, ranging from images of uncentered whole child bodies to properly aligned lung images (Figure 2). To aid the paediatric pneumonia prediction task, we first aim to identify the location of the lung. While techniques such as data augmentation can minimize the impact of translation, scaling, and rotation on a neural network's performance, explicitly removing these sources of variation can improve performance [45]. We will evaluate two approaches for their ability to identify the lungs location within an X-ray image. The first model's architecture is a variant of the VGG (Visual Geometry Group) architecture, but with residual network blocks for pixel-level object detection. This has previously been shown to work well for adult lung segmentation [44,46], in combination with image histogram equalization. This architecture of this lung detection model will be determined from a hyper-parameter search, which will match the model's complexity to the lung detection problem. The lung detection model will initially be trained on 300 paediatric chest X-ray images and evaluated on a validation set of an additional 100 paediatric chest X-ray images and assessed using the Dice coefficient to evaluate the model's performance. Additional images may be used if the model's performance is deemed insufficient. In line with observations by Dai and colleagues [44], 300 images should be sufficient for our lung segmentation and centering task. Key point detection on the segmented images by Binary Robust Invariant Scalable Keypoints (BRISK) [47] or Fast Retina Keypoint (FREAK) [48] to align the images to a reference image using the Open Source Computer Vision Library (OpenCV), an image manipulation toolkit [49]. The second approach for lung detection will be to explore the use of spatial transformer networks [45] in conjunction with object recognition deep learning architectures, thereby enabling a single model to detect the lung and diagnose paediatric pneumonia.

For peer review only

The second stage of the software tool will diagnose pneumonia from the aligned paediatric chest X-ray images. This identification task will be performed in a multi-task prediction setting, namely that the CNN's goal will be to predict 1) primary end-point pneumonia, 2) other lung infiltrates, and/or 3) pleural fluid in both the left and right lung for a total of 6 binary labels. The pneumonia diagnosis model will be based on the DenseNet architecture [25]. Dilated convolutions [50] will be incorporated into the architecture to aggregate features spanning different distances across the lungs. The final model architecture will be determined from an architecture hyper parameter search over the number of convolution layers per dense block, feature-map growth rate, and feature-map compression will be explored. The number of dense blocks will be kept fixed to four. The binary cross entropy loss function will be used for model training, to assess differences between the model's prediction and the ground truth provided by the labels and to guide how the model parameters are to be updated to improve the model predictions. An ensemble of five models will form the final predictor, whose predictions for each of the 6 output classes will be the unweighted average of the five models' predicted class probabilities.

Statistical Methods and Performance Evaluation

To evaluate the quality of the labels associated with the ~10,000 chest X-ray images, we will evaluate the readers' concordance with each other and with the compiled multi-reader labels using Cohen's kappa coefficient, sensitivity, and specificity for both the right and left lung. 95% confidence intervals will be assessed empirically by bootstrapping.

To select the model hyper-parameters (model complexity), the data will be partitioned into 5-folds and tested on 10% of the data. This will allow us to train models on 72% (4 folds) and validate on 18% (1 fold) of the data and to compare models with different hyper-parameters. Given the prevalence of pneumonia within this dataset measured to date (29%), having enough positive samples to validate the model for both the right and left lung is a concern, which we believe a 5-fold cross validation addresses. Hyper-parameters will be determined with a mixture of grid searching and prior literature values. These five models will form an ensemble

For peer review only

and the final predictions for each of the 6 output classes will be formed by taking the unweighted average of the predicted class probabilities for each label.

Two external image datasets (objective 3) and 10% of the data derived from primary 10,000 image dataset will be used to evaluate the software tool. The first external test set will be from KWMCH, whose labels will be provided by the sa me readers who labelled the training image dataset. This dataset will enable the study to evaluate the impact of using different X-ray imagining machines and technicians and disease prevalence. The second external test dataset will be from WHO CRES, whose images were independently collected and independently labelled by clinicians and radiologists following the WHO Radiological Pneumonia definitions [4]. This will assess how transferable our method will be to other study sites. Performance will be evaluated using the area under the receiver operator curve (auROC), the area under the precision-recall curve (auPR), sensitivity, and specificity. A confusion matrix will also be provided. Finally, 95% confidence intervals will be assessed empirically by bootstrapping. Benchmark dataset will be resampled with replacement 1000 times.

Patient and Public Involvement

No patient or members of the public were involved in this study.

DISCUSSION

The study will be the first to use a large dataset whose labels are derived from multiple expert readers to train a deep-learning model to diagnose paediatric primary-end point pneumonia. Prior work to identify pneumonia in adult lungs using a CNN achieved a performance of 0.931 auROC and a 0.815 auROC on an independently collected benchmark. This computational model was trained on chest X-ray images labelled from a single reader whose pneumonia labels were extracted from radiology reports via a NLP system that may introduce extraction errors [12]. A prior study that leverage multiple expert readers, was small in size, containing 858

For peer review only

pediatric chest X-ray images interpreted for PEP by three readers [2]. Since the X-rays were collected over a short period of time, this dataset likely lacks the image variance observed in LMIC settings. The study authors used a SVM to make pixel level predictions and a 95th percentile pixel score as an image score to achieve a 0.850 auROC [13]. We are expecting our model to have similar or better performance since the training data is larger in size and evaluated by multiple readers. Furthermore, the radiological pneumonia definition is broad in comparison to the more precisely defined WHO of diagnosing primary-end point pneumonia.

A limitation of the study is the use of chest X-ray images from individuals ≤ 60 months of age derived from a single institution using two machines to capture the images to construct a computational model to interpret chest X-ray images for the presence of pneumonia. Findings from the study may not generalize to individuals ≥60 months of age. However, this is the most appropriate age group for this study as more than 90% of pediatric pneumonia cases are limited to children <5 years of age.

While the computational model will be evaluated against an independently collected and labelled chest X-ray image dataset for the presence or absence of primary end-point pneumonia (WHO CRES dataset), some findings may not generalize to other settings (as the training data is from a single institution). To overcome this limitation, we are aiming to extend this study as a multi-country surveillance, if successful.

For peer review only

Data Availability

All findings will be published in an open-access journal. All X-ray labels and statistical code will be made openly available. The model and images will be made available upon request.

Acknowledgements

This research was commissioned by the UK National Institute for Health Research (NIHR) Global Health Research Unit on Respiratory Health (RESPIRE), using UK Aid from the UK Government. The views expressed in this publication are those of the author(s) and not necessarily those of the NIHR or the UK Department of Health and Social Care. We are grateful to Mohammad Shahidul Islam and Md. Shariful Islam for the intellectual and technical guidance.

Author's Contributors

Conceptual construction of research and design: MGFS, SS, and SKS. Critical manuscript review: MGFS, SS, SKS, SAS, SL and HN. All authors have approved the final version of the manuscript for submission for publication.

Funding

This work has received funding support from NIHR Global Health Research Unit on Respiratory Health (RESPIRE) 16/136/109.

Competing interests

None declared

For peer review only

Ethics and Dissemination

All study protocols were approved by the Ethical Review Committees of the Bangladesh Institute of Child Health, Bangladesh. The study sponsor deemed it unnecessary to attain ethical approval from the Academic and Clinical Central Office for Research and Development of University of Edinburgh, UK. The study uses existing X-ray images from an ongoing WHO-coordinated surveillance. All findings will be published in an open-access journal. All X-ray labels and statistical code will be made openly available. The model and images will be made available upon request.

References

1 McAllister DA, Liu L, Shi T, *et al.* Global, regional, and national estimates of pneumonia morbidity and mortality in children younger than 5 years between 2000 and 2015: a systematic analysis. *Lancet Glob Health* 2019;**7**:e47–57.

2 O'Brien KL, Baggett HC, Brooks WA, *et al.* Causes of severe pneumonia requiring hospital admission in children without HIV infection from Africa and Asia: the PERCH multi-country case-control study. *The Lancet* 2019.

3 National Institute of Population Research and Training. BANGLADESH DEMOGRAPHIC AND HEALTH SURVEY. 2014.https://dhsprogram.com/pubs/pdf/FR311/FR311.pdf

4 Cherian T, Mulholland EK, Carlin JB, *et al.* Standardized interpretation of paediatric chest radiographs for the diagnosis of pneumonia in epidemiological studies. *Bull World Health Organ* 2005;**83**:353–9.

5 Elemraid MA, Muller M, Spencer DA, *et al.* Accuracy of the interpretation of chest radiographs for the diagnosis of paediatric pneumonia. *PLoS One* 2014;**9**:e106051.

6 McCollum ED, Ahmed S, Chowdhury NH, *et al.* Chest radiograph reading panel performance in a Bangladesh pneumococcal vaccine effectiveness study. *BMJ Open Respir Res* 2019;**6**:e000393.

7 Liu Y, Gadepalli K, Norouzi M, *et al.* Detecting cancer metastases on gigapixel pathology images. *ArXiv Prepr ArXiv170302442* 2017.

8 McKinney SM, Sieniek M, Godbole V, *et al.* International evaluation of an AI system for breast cancer screening. *Nature* 2020;**577**:89–94.

9 Lakhani P, Sundaram B. Deep learning at chest radiography: automated classification of pulmonary tuberculosis by using convolutional neural networks. *Radiology* 2017;**284**:574–82.

For peer review only

10 Gulshan V, Peng L, Coram M, *et al.* Development and validation of a deep learning algorithm for detection of diabetic retinopathy in retinal fundus photographs. *Jama* 2016;**316**:2402–10.

11 Rajpurkar P, Irvin J, Zhu K, *et al.* Chexnet: Radiologist-level pneumonia detection on chest x-rays with deep learning. *ArXiv Prepr ArXiv171105225* 2017.

12 Zech JR, Badgeley MA, Liu M, *et al.* Variable generalization performance of a deep learning model to detect pneumonia in chest radiographs: A cross-sectional study. *PLoS Med* 2018;**15**:e1002683.

13 Mahomed N, van Ginneken B, Philipsen RHHM, *et al.* Computer-aided diagnosis for World Health Organization-defined chest radiograph primary-endpoint pneumonia in children. *Pediatr Radiol* 2020;**50**:482–91. doi:10.1007/s00247-019-04593-0

14 Rajpurkar P, Irvin J, Ball RL, *et al.* Deep learning for chest radiograph diagnosis: A retrospective comparison of the CheXNeXt algorithm to practicing radiologists. *PLoS Med* 2018;**15**:e1002686.

15 Russakovsky O, Deng J, Su H, *et al.* Imagenet large scale visual recognition challenge. *Int J Comput Vis* 2015;**115**:211–52.

16 Shin H-C, Roth HR, Gao M, *et al.* Deep convolutional neural networks for computer-aided detection: CNN architectures, dataset characteristics and transfer learning. *IEEE Trans Med Imaging* 2016;**35**:1285–98.

17 Rajaraman S, Candemir S, Kim I, *et al.* Visualization and interpretation of convolutional neural network predictions in detecting pneumonia in pediatric chest radiographs. *Appl Sci* 2018;**8**:1715.

18 Stephen O, Sain M, Maduh UJ, *et al.* An efficient deep learning approach to pneumonia classification in healthcare. *J Healthc Eng* 2019;**2019**.

19 Kermany DS, Goldbaum M, Cai W, *et al.* Identifying medical diagnoses and treatable diseases by image-based deep learning. *Cell* 2018;**172**:1122–31.

20 Rahman T, Chowdhury ME, Khandakar A, *et al.* Transfer learning with deep convolutional neural network (CNN) for pneumonia detection using chest X-ray. *Appl Sci* 2020;**10**:3233.

21 Ucar F, Korkmaz D. COVIDiagnosis-Net: Deep Bayes-SqueezeNet based diagnosis of the coronavirus disease 2019 (COVID-19) from X-ray images. *Med Hypotheses* 2020;**140**:109761.

22 Chouhan V, Singh SK, Khamparia A, *et al.* A novel transfer learning based approach for pneumonia detection in chest X-ray images. *Appl Sci* 2020;**10**:559.

For peer review only

23 Hashmi MF, Katiyar S, Keskar AG, *et al.* Efficient pneumonia detection in chest xray images using deep transfer learning. *Diagnostics* 2020;**10**:417.

24 Krizhevsky A, Sutskever I, Hinton GE. Imagenet classification with deep convolutional neural networks. *Commun ACM* 2017;**60**:84–90.

25 Huang G, Liu Z, Van Der Maaten L, *et al.* Densely connected convolutional networks. In: *Proceedings of the IEEE conference on computer vision and pattern recognition*. 2017. 4700–8.

26 Szegedy C, Vanhoucke V, Ioffe S, *et al.* Rethinking the inception architecture for computer vision. In: *Proceedings of the IEEE conference on computer vision and pattern recognition*. 2016. 2818–26.

27 Szegedy C, Liu W, Jia Y, *et al.* Going deeper with convolutions. In: *Proceedings of the IEEE conference on computer vision and pattern recognition*. 2015. 1–9.

28 He K, Zhang X, Ren S, *et al.* Deep residual learning for image recognition. In: *Proceedings of the IEEE conference on computer vision and pattern recognition*. 2016. 770–8.

29 Vaswani A, Shazeer N, Parmar N, *et al.* Attention is all you need. *ArXiv Prepr ArXiv170603762* 2017.

30 Cha S-M, Lee S-S, Ko B. Attention-Based Transfer Learning for Efficient Pneumonia Detection in Chest X-ray Images. *Appl Sci* 2021;**11**:1242.

31 Toğaçar M, Ergen B, Cömert Z, *et al.* A deep feature learning model for pneumonia detection applying a combination of mRMR feature selection and machine learning models. *Irbm* 2020;**41**:212–22.

32 Zhao W, Wang L, Zhang Z. Artificial ecosystem-based optimization: a novel nature-inspired meta-heuristic algorithm. *Neural Comput Appl* 2019;:1–43.

33 Sahlol AT, Abd Elaziz M, Tariq Jamal A, *et al.* A novel method for detection of tuberculosis in chest radiographs using artificial ecosystem-based optimisation of deep neural network features. *Symmetry* 2020;**12**:1146.

34 Sahlol AT, Yousri D, Ewees AA, *et al.* COVID-19 image classification using deep features and fractional-order marine predators algorithm. *Sci Rep* 2020;**10**:1–15.

35 Zhang J, Xie Y, Pang G, *et al.* Viral Pneumonia Screening on Chest X-rays Using Confidence-Aware Anomaly Detection. *IEEE Trans Med Imaging* 2020.

36 Ke Q, Zhang J, Wei W, *et al.* A neuro-heuristic approach for recognition of lung diseases from X-ray images.

For peer review only

37 Mirjalili S. Moth-flame optimization algorithm: A novel nature-inspired heuristic paradigm. *Knowl-Based Syst* 2015;**89**:228–49.

38 Mirjalili S. The ant lion optimizer. *Adv Eng Softw* 2015;**83**:80–98.

39 WHO Chest Radiography in Epidemiological Studies (CRES). https://who-cres.mcri.edu.au/

40 Mahomed N, Fancourt N, De Campo J, *et al.* Preliminary report from the World Health Organisation chest radiography in epidemiological studies project. *Pediatr Radiol* 2017;**47**:1399–404.

41 Hasan AZ, Saha S, Saha SK, *et al.* Using pneumococcal and rotavirus surveillance in vaccine decision-making: A series of case studies in Bangladesh, Armenia and the Gambia. *Vaccine* 2018;**36**:4939–43. doi:10.1016/j.vaccine.2018.06.001

42 Global Invasive Bacterial Vaccine-Preventable Diseases Surveillance — 2008–2014. https://www.cdc.gov/mmwr/preview/mmwrhtml/mm6349a4.htm (accessed 2 Sep 2020).

43 Saha SK, Hossain B, Islam M, *et al.* Epidemiology of invasive pneumococcal disease in Bangladeshi children before introduction of pneumococcal conjugate vaccine. *Pediatr Infect Dis J* 2016;**35**:655–61.

44 Dai W, Dong N, Wang Z, *et al.* Scan: Structure correcting adversarial network for organ segmentation in chest x-rays. In: *Deep Learning in Medical Image Analysis and Multimodal Learning for Clinical Decision Support*. Springer 2018. 263–73.

45 Jaderberg M, Simonyan K, Zisserman A, *et al.* Spatial transformer networks. In: *Advances in neural information processing systems*. 2015. 2017–25.

46 Bansal A, Chen X, Russell B, *et al.* Pixelnet: Towards a general pixel-level architecture. *ArXiv Prepr ArXiv160906694* 2016.

47 Leutenegger S, Chli M, Siegwart RY. BRISK: Binary robust invariant scalable keypoints. In: *2011 International conference on computer vision*. Ieee 2011. 2548–55.

48 Alahi A, Ortiz R, Vandergheynst P. Freak: Fast retina keypoint. In: *2012 IEEE Conference on Computer Vision and Pattern Recognition*. Ieee 2012. 510–7.

49 Bradski G. The OpenCV Library. *Dr Dobbs J Softw Tools* 2000.

50 Yu F, Koltun V. Multi-scale context aggregation by dilated convolutions. *ArXiv Prepr ArXiv151107122* 2015.

For peer review only

FIGURE LEGENDS

Figure 1: Study design schema. PEP: Primary end point pneumonia; KWMCH : Kumudini Women's Medical College Hospital

Figure 2: Digitized chest X-ray images include (Left) full body images and (Right) images of just the lung area.

For peer review only

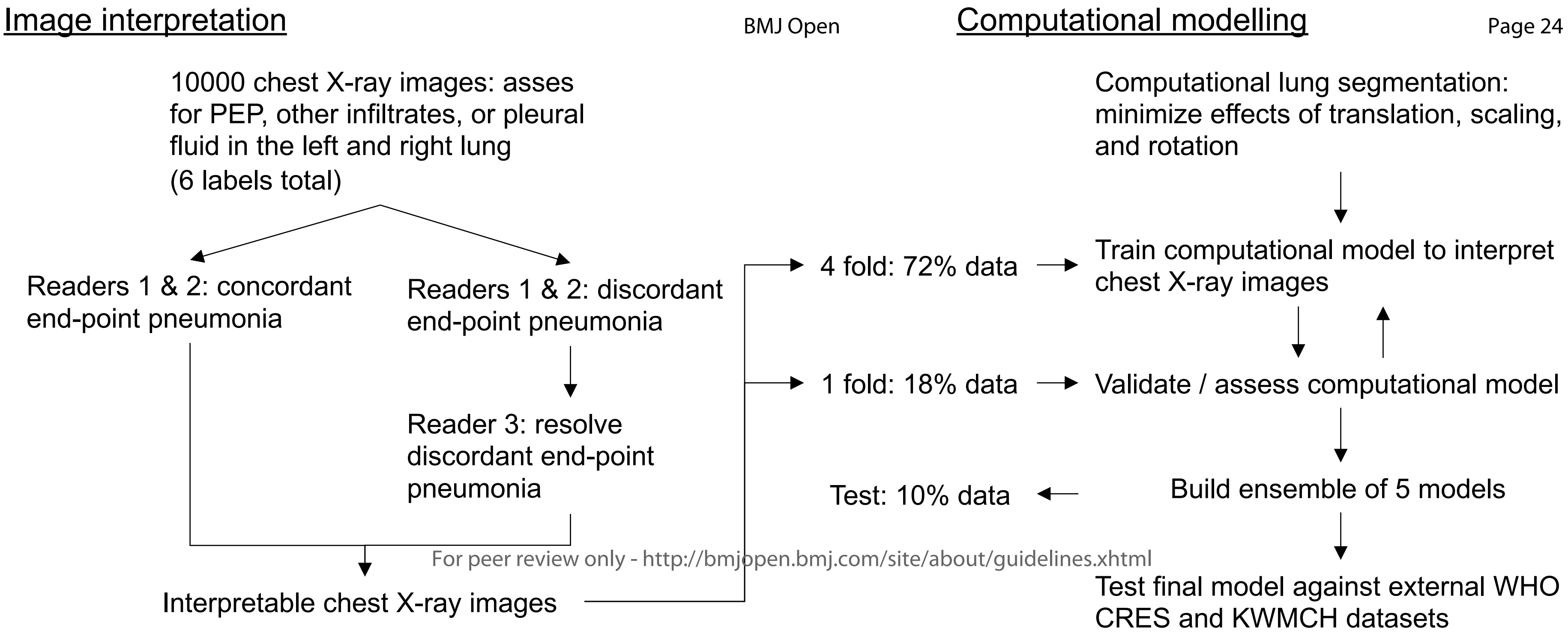

For peer review only - http://bmjopen.bmj.com/site/about/guidelines.xhtml

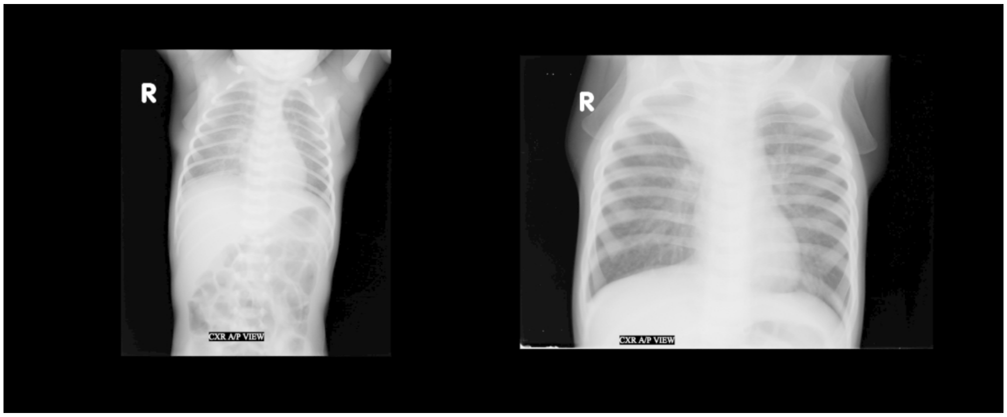

Figure 2: Digitized chest X-ray images include (Left) full body images and (Right) images of just the lung area.
